# ABC Transporters at the Blood–Brain Interfaces, Their Study Models, and Drug Delivery Implications in Gliomas

**DOI:** 10.3390/pharmaceutics12010020

**Published:** 2019-12-23

**Authors:** David Gomez-Zepeda, Méryam Taghi, Jean-Michel Scherrmann, Xavier Decleves, Marie-Claude Menet

**Affiliations:** 1 Inserm, UMR-S 1144, Optimisation Thérapeutique en Neuropsychopharmacologie, 75006 Paris, France; meryam.taghi@parisdescartes.fr (M.T.); jean-michel.scherrmann@parisdescartes.fr (J.-M.S.); xavier.decleves@parisdescartes.fr (X.D.); 2 Sorbonne Paris Cité, Université Paris Descartes, 75006 Paris, France; 3 Sorbonne Paris Cité, Université Paris Diderot, 75013 Paris, France; 4 UF Biologie du médicament et toxicologie, Hôpital Cochin, AP HP, 75006 Paris, France; 5 UF Hormonologie adulte, Hôpital Cochin, AP HP, 75006 Paris, France

**Keywords:** ABC transporters, blood–brain barrier (BBB), blood–cerebrospinal fluid barrier (BCSFB), arachnoid barrier (BAB), blood–brain tumor barrier (BBTB), glioma, drug delivery

## Abstract

Drug delivery into the brain is regulated by the blood–brain interfaces. The blood–brain barrier (BBB), the blood–cerebrospinal fluid barrier (BCSFB), and the blood–arachnoid barrier (BAB) regulate the exchange of substances between the blood and brain parenchyma. These selective barriers present a high impermeability to most substances, with the selective transport of nutrients and transporters preventing the entry and accumulation of possibly toxic molecules, comprising many therapeutic drugs. Transporters of the ATP-binding cassette (ABC) superfamily have an important role in drug delivery, because they extrude a broad molecular diversity of xenobiotics, including several anticancer drugs, preventing their entry into the brain. Gliomas are the most common primary tumors diagnosed in adults, which are often characterized by a poor prognosis, notably in the case of high-grade gliomas. Therapeutic treatments frequently fail due to the difficulty of delivering drugs through the brain barriers, adding to diverse mechanisms developed by the cancer, including the overexpression or expression de novo of ABC transporters in tumoral cells and/or in the endothelial cells forming the blood–brain tumor barrier (BBTB). Many models have been developed to study the phenotype, molecular characteristics, and function of the blood–brain interfaces as well as to evaluate drug permeability into the brain. These include in vitro, in vivo, and in silico models, which together can help us to better understand their implication in drug resistance and to develop new therapeutics or delivery strategies to improve the treatment of pathologies of the central nervous system (CNS). In this review, we present the principal characteristics of the blood–brain interfaces; then, we focus on the ABC transporters present on them and their implication in drug delivery; next, we present some of the most important models used for the study of drug transport; finally, we summarize the implication of ABC transporters in glioma and the BBTB in drug resistance and the strategies to improve the delivery of CNS anticancer drugs.

## 1. Introduction

Drug delivery and clearance in the central nervous system (CNS) are restricted and regulated by the blood–brain barrier (BBB), the blood–cerebrospinal fluid barrier (BCSFB), and the blood–arachnoid barrier (BAB). These barriers present several mechanisms that are used to regulate the exchange of substances between the blood and the brain, including a high impermeability to most substances and the selective transport of nutrients and transporters, preventing the entry of toxic molecules, comprising many xenobiotics and also therapeutic drugs [1,2,3]. This last function is performed mainly by transporters from the ATP-binding cassette (ABC) superfamily [3], and to a lesser extent, the solute carrier (SLC) superfamily exchangers [1], both of which have a key role in the absorption, distribution, metabolism, and excretion (ADME) of drugs. The ABC transporters are particularly important because they extrude many xenobiotics of a broad molecular variety, including several anticancer drugs, preventing thus their entry to the brain and to the tumors in patients. Therefore, many models of the brain barriers have been developed to study drug transport and delivery into the CNS [4,5,6,7,8].

Cancers of the CNS, and particularly gliomas, represent a worldwide problem for healthcare because patients become highly disabled by the disease, treatments are expensive, and prognosis is poor, due to the tumor’s aggressiveness and resistance to multiple chemotherapeutic drugs [9,10,11]. As in other cancers, brain tumors can present modifications in the DNA repair system and the cell cycle, an enhanced metabolism of xenobiotics, and anti-apoptosis phenotypes [12,13]. In addition, drug delivery and accumulation into brain tumors is restricted by the blood–brain interfaces [14]. Importantly, the overexpression of ABC transporters at the BBB or the blood–brain tumor barrier (BBTB), as well as in the tumor cells, is often observed [15], which can lead to an improved multidrug resistance [16]. Thus, it is important to understand the function of these transporters and their changes in the pathology.

In this review, we present the principal characteristics of the blood–brain interfaces implicated in drug delivery. Then, we focus on the ABC transporters present on these barriers and their implication in drug delivery. Next, we present some of the most important models used for the study of drug transport. Finally, we summarize the implication of ABC transporters in glioma and the BBTB in drug resistance and the strategies to improve the delivery of CNS anticancer drugs.

## 2. Brain Barriers and Their Implication in Drug Delivery

**Figure 1:**
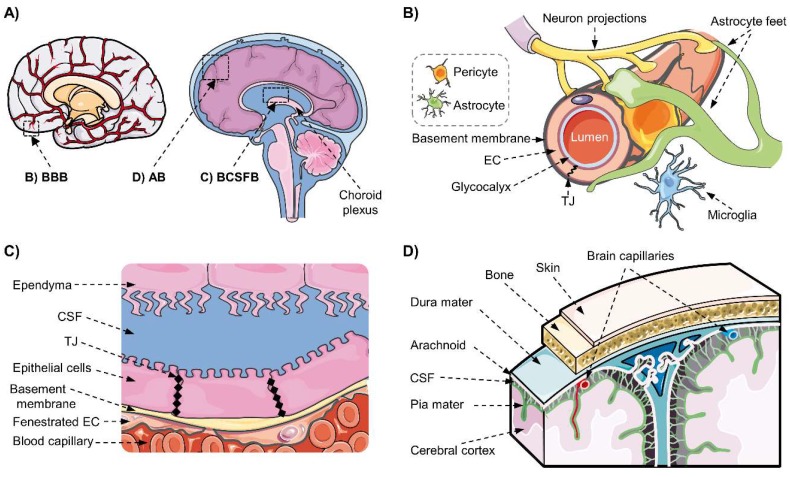
Human blood–brain interfaces. (**A**) There are three main interfaces regulating the exchanges between blood and brain (A), left), either directly to the parenchyma or through the cerebrospinal fluid (CSF; A), right). (**B**) The blood–brain barrier (BBB) is formed mainly by the brain microvascular endothelial cells (EC), attached by tight junctions (TJ), but their specialized phenotype and function are regulated and maintained by the neurovascular unit (NVU) formed by the basement membrane and neighboring cells including pericytes, astrocytes, neurons, and microglia. (**C**) The blood–cerebrospinal fluid barrier (BCSFB) is formed by the tightly jointed epithelial cells of the choroid plexus (CP), which cover the fenestrated EC of the CP capillaries. (**D**) The meninges are composed of three layers: the outermost fibrous sheet of dura mater, the arachnoid mater and the pia, both enclosing CSF in the subarachnoid space; the arachnoid cells present tight junctions and form the blood–arachnoid barrier (BAB). Created using images from “smart Servier Medical Art”, Creative Commons License, 2019.

Three different barriers formed by endothelial or epithelial cells with tight junctions regulate the substance exchange between brain and blood (Figure 1): (1) The blood–brain barrier (BBB) is comprised by the specialized brain microvascular endothelial cells (BMVEC) separating the blood and brain parenchyma and interstitial fluid. (2) The blood–cerebrospinal fluid barrier (BCSFB) is formed by the epithelium of the choroid plexus (CP), which secretes the cerebrospinal fluid (CSF) into the ventricular system and the meninges. (3) The arachnoid barrier (BAB) surrounding the CNS is an avascular multilayered epithelium separating the blood from the subarachnoid CSF. These interfaces act as selective barriers, regulating the entry and distribution of diverse molecules into the brain and their excretion, including medicinal drugs [1,17,18]. Therefore, it is important to study the implication of these barriers in drug delivery to the CNS and pharmacokinetics. The physiology and function of the blood–brain interfaces, as well as their implication in drug delivery, have been extensively reviewed in previous publications [1,2,14,17,18,19]; thus, they will be briefly explained in this section.

### 2.1. The Blood–Brain Barrier and the Neurovascular Unit

The BBB is formed mainly by the brain microvascular endothelial cells (BMVEC), which constitute a physical, transport selective and metabolic barrier. They form a tight monolayer lacking fenestration due to especially tight junctions (TJs) between the cells, restricting the paracellular movement of small polar substances and macromolecules [1,18]. The BMVEC express several transporters with polarized localization at the luminal and/or basolateral membranes to specifically regulate the influx and efflux of molecules, such as nutrients, waste metabolites, toxins, xenobiotics, and small peptides. The main transporter proteins expressed at the BBB are SLC transporters and active efflux pumps from the ABC superfamily [3,20]. The exchange of macromolecules (i.e., larger peptides and proteins) is regulated by limited transendothelial vesicular trafficking. In addition, BMVEC express specialized enzymes for the degradation of multiple substrates including cytochromes P450 (CYPs450), monooxygenases (phase I enzymes), monoamine oxidase, glutathione-S-transferases (GST), methyltransferases, UDP-glucuronosyltransferases (UGT), and methyltransferases (phase II enzymes) as the catechol-O-methyltransferase (COMT) [21,22,23]. Therefore, the capability of a molecule to cross the endothelium depends on its physicochemical properties (such as charge state, hydrophobicity, molecular size, spatial conformation), the concentration gradient, its binding to plasma proteins, transporter affinity, and metabolic processing [6].

The BBB function depends on the dynamic interaction between the BMVEC and its surrounding environment, the neurovascular unit (NVU). The endothelial cells are dynamically regulated by their interactions with the basement membrane surrounding the capillaries, with neighboring cells including pericytes, astrocytes, neurons, and microglia; but also with circulating cells, such as leucocytes, through the glycocalyx on the luminal membrane of endothelial cells (Figure 1B). They maintain a constant communication by direct cell-to-cell interactions, modulations of the extracellular matrix, and the exchanges of soluble factors. This complex and dynamic structure is known as the NVU, whose components are indispensable for the acquisition of the BMVEC phenotype and the maintaining of the BBB functions [4,6]. In addition, it has been observed that the glycocalyx acts as a barrier to large molecules, while the basement membrane and astrocyte endfeet further hinder the entry of small and large molecules into the brain parenchyma, contributing directly to the brain function of the BBB [24].

The BBB is often considered the most important brain barrier for drug delivery. Although some neurotherapeutics, including chemotherapeutics, are now administered by intralumbar injection into the CSF of the subarachnoid space (intrathecally) [25] and there have been tests using intracranial drug administration [26], intravenous injection is still the main way for drug delivery in CNS diseases. The BMVEC forming the BBB comprise the largest exchange interface between blood and parenchyma, with a total area between 12 and 18 m^2^ in the average human adult [27]. Nevertheless, their selective permeability constitutes an obstacle for drug entry into the brain [3,6,18]; the tight junctions block the passage of molecules at the intracellular space [28]; hydrophobic therapeutics that would normally diffuse through the membranes are effluxed by the highly unspecific ABC transporters [3,20]; meanwhile, those that enter into the endothelial cells are inactivated by the battery of metabolic enzymes mentioned above [21,22] before being effluxed [23].

### 2.2. The Blood–Cerebrospinal Fluid Barrier

The choroid plexus (CP) is composed of capillaries formed by fenestrated endothelial cells, enveloped by a basement membrane and a monolayer of tightly jointed epithelium which form the blood–cerebrospinal fluid barrier (BCSFB), as the CSF is enclosed between this layer and a layer of ependyma [19]. The endothelial cells of the choroid plexus are fenestrated and do not present tight junctions; thus, they do not form a barrier for small molecules, but the exchange of substances between blood and CSF is precisely controlled by the tightly jointed epithelial cells at the apical CSF-facing side, forming the BCSFB (Figure 1C), while the exchange between the CSF and the brain is facilitated by the non-jointed ependymal cells. The transport of molecules is selectively regulated by proteins specifically expressed at the luminal and/or basolateral membranes of the epithelial cells, including ABC and SLC [17,19]. The BCSFB also expresses diverse metabolizing enzymes that inactivate endogenous and exogenous molecules, such as CYPs450, GSTs, and UGTs [29,30]. Additionally, the epithelial cells present infoldings on the basolateral membrane and microvilli on the apical membrane, increasing the transfer surface area to improve fluid secretion [31]. Thus, the BCSFB forms a physical, transport, and metabolic barrier controlling the exchanges between blood in the fenestrated capillaries and CSF to protect the brain from possibly toxic substances, but also affecting the entry of therapeutic molecules; thus, its role must be considered when evaluating drug pharmacokinetics [18].

### 2.3. The Blood–Arachnoid Barrier

**Figure 2:**
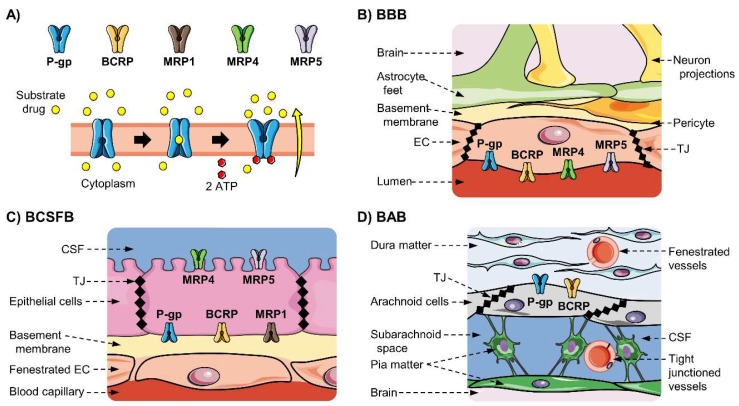
Efflux mechanism and localization of drug-related ATP-binding cassette (ABC) transporters at the human blood–brain interfaces: (**A**) (top) ABC transporters P-glycoprotein (P-gp/MDR1), breast-cancer resistance protein (BCRP), multidrug-resistance proteins 1, 4 and 5 (MRP1, MRP4 and MRP5) detected at the protein level at the blood–brain interfaces in non-pathological human brain; and (**A**) (bottom) simplified schema of their active transport mechanism, where substrates are effluxed against the concentration gradient in an ATP-dependent manner. Schemas showing the polarized localization of ABC transporters at the (**B**) blood–brain barrier (BBB), (**C**) the blood–cerebrospinal fluid barrier (BCSFB) at the choroid plexus and (**D**) the arachnoid barrier (BAB) at the meninges. EC, endothelial cells; TJ, tight junctions; CSF, cerebrospinal fluid. Created using images from “smart Servier Medical Art”, Creative Commons License, 2019.

The arachnoid cells present in the middle layer of the meninges covering the brain and spinal cord constitute the blood–arachnoid barrier (BAB). The meninges are composed of three layers; starting with the outermost pachymeninx composed of a fibrous sheet of dura mater; then, the two innermost layers form the leptomeninges, including the arachnoid mater followed by the pia mater lining the brain, both enclosing CSF in the subarachnoid space [32] (Figure 1D). Blood vessels within the dura mater are fenestrated, but blood vessels in the subarachnoid space present tight junctions. While pia cells do not present tight junctions, the arachnoid cells are tight-junctioned (Figure 2D), and recent studies in human, mouse, and rat models have shown that the BAB cells express metabolizing enzymes such as CYPs450 as well as ABC and SLC transporters specifically localized at the apical and/or basolateral membranes to regulate the passage of substances between the CSF and the fenestrated blood capillaries in the dura matter, acting as a selective barrier [33,34,35].

There has been a growing interest in the role of the BAB in drug delivery to the CNS and its clearance. As the BAB is avascular and presents a small exchange area compared to the BCSFB, its contribution to the blood–brain exchange is often neglected [36]. Nevertheless, its role in drug influx and efflux at the CNS could be more important than thought [32], considering their barrier phenotype. The amount of unbound drug in the interstitial fluid in the brain is often assessed using its concentration in the CSF as a surrogate [37], but this may differ due to drug clearance into the capillaries in the dura matter performed by transporters present in the BAB cells [33,34]. Furthermore, some chemotherapies and other drugs are administered through intralumbar injection, intrathecally into the CSF of the subarachnoid space [25]. For instance, it is used to treat meningitis, leptomeningeal tumors, and particularly CSF lymphoblasts in childhood acute lymphoblastic leukemia patients [38]. Therefore, it is important to consider that the entry and distribution of intrathecally administered drugs depend on their passage across the BAB cells, which present a similar phenotype to the BBB and thus can metabolize and efflux drugs from the CSF to the fenestrated capillaries through phase I and phase II enzymes and ABC transporters, respectively [33,34,35].

### 2.4. Conclusion on the Blood–Brain Interfaces and Their Implication in Drug Delivery

Together, the three blood–brain interfaces contribute to the selective permeability and clearance of substances in and out of the CNS to maintain its homeostasis. Their barrier function is performed by orchestrated mechanisms such as tight junctions to form physical barriers, selective permeability through passive and active transport mechanisms, and biochemical protection using specialized enzymes to degrade toxic molecules. The active transporters of the ABC superfamily are major gatekeepers of these interfaces, which selectively effluxes a high diversity of molecules, including xenobiotics and many chemotherapeutics, as it will be further explained in the next section.

## 3. Drug-Related ABC Transporters and Their Role at the Blood–Brain Interfaces

### 3.1. The ABC Superfamily

The ATP-binding cassette (ABC) superfamily groups many membrane proteins that act as active efflux pumps of many substances, including therapeutics. ABC transporters comprise a highly conserved “cassette-like” domain that catalyzes the ATP hydrolysis providing the energy needed for the transport of substances against a concentration gradient [39] (Figure 2A). They actively transport both endogenous and exogenous substances and are implicated in the absorption, distribution, and excretion of several xenobiotics [39,40,41,42]. To date, 48 ABC genes and three pseudogenes can be found in humans and, according to the HUGO Gene Nomenclature Committee [43], they are grouped in seven subfamilies named with the letters A–G (i.e., ABCA, ABCB, …, ABCG).

Due to their strategic tissue localization and low substrate specificity, the ABC transporters are fundamental for the protection from toxic substances, transport of important metabolites, and cell signaling. ABC transporters are found in the intestine, liver, kidney, hearth, lungs, brain, placenta, and testis. More particularly, they are expressed in the tissue interfaces such as endothelia and blood–tissue barriers; where they are located in the cellular plasma membrane, acting as efflux pumps of toxic molecules; or in the Golgi apparatus, mitochondria, or endoplasmic reticulum avoiding intra-organelle toxicity [44,45]. In the blood–brain interfaces, ABC transporters are located mostly in the luminal barriers [16,20,33,34,46], pumping out substances in a brain-to-blood sense; although members of the ABCC subfamily are located at the basolateral membrane of the CP [20,47]. Each ABC transporter can have a wide spectrum of substrates, which can include amino acids, sugars, peptides, diverse hydrophobic compounds, and their metabolites; and thus, diverse drugs of these characteristics. Therefore, they have a key role in the regulation of drug delivery into the central nervous system and an important impact in their pharmacology effects [36,48,49]. In this section, the ABC transporters with an important function in CNS drug delivery (Table 1), and their localization in the blood–brain interfaces (Table 2, Figure 2B–D)) will be reviewed.

### 3.2. ABC Transporters Related to Drug Transport

The ABCB, ABCC and ABCG subfamilies include transporters related to xenobiotics efflux and drug resistance, notably ABCB1 (P-gp), ABCG2 (BCRP), and the multidrug resistance subfamily (MRPs) of ABCCs [36,56]. Due to the broad spectrum of ABC substrates, including many therapeutics (Table 1), they play an important role in drug ADME. In addition, their expression can be modulated as a cause or a part of pathological states such as epilepsy [97] and many tumors and cancer cells, including brain tumors (Section 5.4) [6,16]. In addition, ABC transporters can present a synergic or complementary function, and it has been observed that they may relay one to another [53,98,99]. This can lead to the development or improvement of multidrug resistance by preventing the drug from reaching the tissue and the molecular target, hampering thus the therapeutic effect. The function of ABC transporters in drug transport will be briefly summarized below, with a focus on anticancer therapeutics.

The P-glycoprotein (P-gp/MDR1) was the first ABC transporter to be associated with multidrug resistance in cancer cell lines [100]. In humans, P-gp is encoded by the *ABCB1* (*MDR1*) gene, while in rodents, two isoforms have been found, *Abcb1a* and *Abcb1b* (Mdr1a and Mdr1b proteins) with different tissue-specific localization [85,101,102]. P-gp can transport a very broad spectrum of molecules with different chemical structure, molecular weight, and properties. It is difficult to define canonical properties of P-gp substrates, but they are typically hydrophobic or amphipathic, with a planar mainly aromatic ring system and positively charged (at physiological pH), although some neutral substrates are also observed [50] (Table 1). It is implicated in the translocation of many anti-cancer drugs including anthracyclines, vinca alkaloids, taxanes, and tyrosine kinase inhibitors (gefitinib, imatinib mesylate) [16,103,104]).

Breast-cancer resistance protein (BCRP) functional form is formed by a homo-oligomer of the 72 KDa polypeptides encoded by the *ABCG2* gene. The structure of the functional protein is still debated but it has been observed that the BCRP polypeptide can be assembled in homodimers, tetramers, or even duodecamers (reviewed in [105]). The list of BCRP substrates is also large (over 200) and diverse; it includes some common substrates with P-gp or the multidrug resistance protein 1 (MRP1/*ABCC1*) [52,53], but also contains a lot of distinct compounds (Table 1). Many of the substrates identified are chemotherapeutics, including camptothecin derivatives (topotecan), anthracyclines (mitoxantrone), polyglutamates (methotrexate), as well as tyrosine kinase inhibitors such as imatinib and gefitinib [105,106]. No structure–activity relationship has been properly identified between the many BCRP substrates, which complicates drug development and screening.

The ABCC subfamily comprise 13 protein-coding genes for humans, while nine are related to the multidrug-resistance protein (MRP) subclass: MRP1-6 (*ABCC1*-*6*), MRP7-9 (*ABCC10*-*12*), and *ABC13*, a non-functional pseudogene [55]. The other three are the sulfonylurea receptors 1 and 2 (SUR1, 2/*ABCC8*, *9*) and the cystic fibrosis transmembrane conductance regulator (*ABCC7*). Each MRP has a specific substrate profile and although overlap can occur, their kinetics are often different [107] (reviewed in [55], Table 1). Generally, MRPs transport a variety of organic anions which can be or not conjugated with glutathione (GSH), glucuronide, sulfate, or phosphate [3]. In some cases, GSH acts as an activator, while in others, it is not needed [55,56,108,109]. Thus, the MRPs work together with metabolism enzymes to efflux endogenous substances as oestradiol 17-β-D-glucuronide (E_2_17βG), the pro-inflammatory cytokine LTC_4_ (leukotriene C_4_), and bile acids. They also extrude xenobiotics, including many anticancer drugs such as doxorubicin, vincristine, etoposide, campathecin, topotecan, and methotrexate [55,56,108,109]. MRP4 and MRP5 can also transport nucleotides as cyclic AMP and cyclic GMP [110] and confers resistance to several antiviral and anticancer nucleotide analogues [111,112,113].

### 3.3. ABC Transporters Expressed at the BBB and the NVU

P-gp/MDR1 (*ABCB1*) is expressed at the luminal (apical) membrane of brain microvessels endothelial cells and is not found in healthy parenchymal cells (Table 2, Figure 2B))**.** P-gp/MDR1 (*ABCB1*) has been detected in the brain endothelial cells of many mammals including humans, rats, mice, bovines, porcines, and other primates by different techniques [22,63,86,114,115], where it is located to the luminal side of the cell [58,59,60,61,62,63]. In mouse and rat, the transcript of *Abcb1a* isoform is predominant in BMVEC, while a low expression of *Abcb1b* was observed [85,101,102]. P-gp expression has been reported in parenchymal cells in culture or from pathological origins, but its function has not been proved in in vivo healthy samples. Functional Mdr1b/MDR1 has been detected in cultured mouse [116], rat [117,118], and human [69] astrocytes as well as cultured rat microglia [119]. However, P-gp expression could be induced by culture conditions [16]. In rat and mouse, low mRNA expression of *Mdr1b* was observed in astrocytes, microglia, and endothelial cells, but this was not confirmed at the protein level [85,101,102]. Furthermore, no local translation of ABC transporters was observed at the mouse astrocytes’ endfeet surrounding the vascular surface [64], where P-gp was believed to be expressed. In humans, it has been detected in parenchymal cells of tissues obtained from patients with epilepsy [62,67,69] or gliomas [66], but the pathology may have induced the P-gp de novo expression, as it was not expressed in the healthy (control) tissue neither at the mRNA [65,66] nor at the protein levels [66,67,68,69].

BCRP (*ABCG2*) is located to the luminal membrane of microvessels endothelial cells, and its expression in parenchymal cells has not been proved (Table 2, Figure 2B)). *ABCG2* mRNA transcript has been detected in human, mouse, rat, and porcine microvessels, where the BCRP protein is located at the luminal membrane [63,78,79,80,81,82,83]. According to the RNA-Seq brain transcriptome, *Abcg2* in mouse [85] and *ABCG2* in humans [65] is expressed mostly in endothelial cells, but low transcript levels could be found in microglia. Abcg2 has also been detected in a pericyte cell line [120] and in primary cultured astrocytes [84]. Immunohistochemistry assays by the Human Protein Atlas indicate a high BCRP expression in the endothelial cells and low in neuropil [68], which includes cell processes from neurons and glial cells, indicating possible expression in parenchymal cells. Nevertheless, the protein expression and function of Bcrp/BCRP in vivo in parenchymal cells needs to be clarified.

MRP4 and MRP5 are expressed at the luminal membrane of brain microvessels endothelial cells in humans, but interspecies differences may occur (Table 2, Figure 2B)**.** Different studies have shown the mRNA expression of *ABCC1* to *ABCC10* in human, rat, mouse, porcine, and bovine models, [21,46,86] with important differences between the species [86]. Nevertheless, the human samples in these studies consisted of perilesional and cortex (far from lesions) biopsies from glioma or epileptic patients undergoing surgical resections, and the expression may be induced by the pathology [6,14,16]. Nevertheless, MRP3 expression at the protein level has not been detected by immunohistochemistry (IHC) [46,86,96,121], and no MRP1 function was observed in mouse microvessels [122], suggesting that they are not expressed at the BBB in basal conditions. In humans, MRP4 and MRP5 proteins are located in the luminal membrane [46]. Some publications have reported the expression of MRP1 in the BBB, but it is very likely that this results from hazardous extrapolations from in vitro data to the in vivo situation, because MRP1 function was not detected in human microvessels from healthy human samples [46] and function was not proven by in situ brain perfusion in mice [122]. Indeed, it has been observed that MRP1 may be overexpressed due to cell culture conditions or the origin of the samples from diseased patients [15,66]. Mrp2 has been observed in the luminal membrane of BMVEC only in rat and mouse [90,91], indicating important differences between the species. MRP4 has been quantified using mass spectrometry (MS) in humans, rodents, marmosets, and monkey isolated brain microvessels [22,92,93,94,95], but it is expressed in very low levels, around 10 to 20-fold lower than P-gp; while other MRPs were under the limit of quantification.

MRP5 is lowly expressed in neuropil and MRP2 in neuropil, glial, and neuronal cells (in human) (Table 2, Figure 2B). mRNA transcripts of *Mrp1*, *Mrp3*, *Mrp4* and *Mrp5* have been observed in primary cultures of rat microglia and oligodendroglia [123], but it is highly likely that expression was triggered by culture conditions or the disease origin of samples. Particularly, it has been suggested that functional MRP1 is expressed in astrocytes, as *Mrp1* mRNA expression and function were observed in rat cultured astrocytes [117], and the MRP1 protein was detected in astrocytes by IHC in tissue slices from glioma human patients, and function was proven using primary cultures [15,66]. Nevertheless, no local translation of *Abcc* transporters was observed at the mouse astrocytes’ endfeet surrounding the vascular surface [64]. MRP4 and MRP5 have been detected in human astrocytes, while MRP5 has also been observed in human pyramidal neurons [46] and astrocytes [15], as well as primary cultures of rat astrocytes [123] and microglia [47,124]; nevertheless, these experiments were performed in samples from patients with epilepsy or glioma, and the expression may be tuned by the disease. In the contrary, the IHC assays reported at the Human Protein Atlas were performed on healthy tissue and better represent the basal expression of proteins. According to this database, in the human brain cortex, MRP1 and MRP4 are not detected in glial or neuronal cells, MRP3 was not detected in the brain cortex, MRP5 was detected in low levels in neuropil, and MRP2 was observed in low levels in glial and neuronal cells, as well as in the neuropil in medium level but not in the endothelial cells [68].

### 3.4. ABC Transporters Expression at the BCSFB

P-gp is lowly expressed at the apical membrane of the CP epithelial cells (Table 2, Figure 2C)). It has been detected in the mRNA and protein levels at the BCSFB of humans and rats (*mdr1a* and *mdr1b*) [70,71,72,73], and it has been located as weakly expressed at the apical side of the human, rat, and mouse CP [33,71,72,73]. Nevertheless, its protein expression in rat was shown to be 77.8-fold lower in the CP than in brain microvessels (0.320 vs. 24.9 fmol/μg_protein_) [70,93]. In humans, P-gp is 1.9 times lower in CP than in brain microvessels (2.10 vs. 3.98 fmol/μg_protein_) [22,70].

BCRP is lowly expressed at the apical membrane of CP epithelial cells (Table 2, Figure 2C). BCRP has been detected in mouse, rat, and human CP [20,33,70,81], and low BCRP expression has been located at the apical side of human and mouse CP [20,33,81]. Although it could not be detected by IHC in rat CP [83], the Bcrp protein has been quantified using LC-MS/MS in rats and humans [70]. Its protein expression in rat was shown to be 21-fold lower in the CP than in brain capillaries (0.330 Vs 6.95 fmol_monomer_/μg_protein_) [70,93]. In humans, BCRP is 8.7 times lower in the CP than in brain microvessels (0.706 Vs 6.15 fmol_monomer_/μg_protein_) [22,70].

MRP1 and MRP4 in humans, rats, and mice, and MRP5 in rats are expressed at the basolateral membrane of the CP epithelial cells (Table 2, Figure 2C). In humans, *MRP1* to *MRP6* have been detected at the mRNA level [87], but only proteins MRP1 and MRP4 have been observed [71,96]. Similarly, in rats, *Mrp1* to *Mrp6* mRNA transcripts have been detected [88], but only proteins Mrp1, Mrp4, and Mrp5 have been observed [70,83]. In mouse, *Mrp1*/Mrp1 and *Mrp4*/Mrp4 were detected at the mRNA and protein level [89,96]. MRP1 and MRP4 have been located at the basolateral side of the CP epithelial cells from humans, mice, and rats [71,73,83,89,96] as well as MRP5 in rat [83]. Interestingly, MRP4 is the only ABC transporter found at the basolateral membrane of the CP epithelium and in the luminal membrane of the BMVEC [96] endothelial cells of the brain capillaries. It has been observed that MRP1 is the ABC transporter with the highest expression at the CP in rats (5.47 fmol/μg_protein_) and the second highest in humans (1.36 fmol/μg_protein_), after P-gp (2.10 fmol/μg_protein_) [70]; additionally, in both cases, MRP1 is higher at the CP than in BMVEC (below the limit of quantification) [22,93].

### 3.5. ABC Transporters Expression at the BAB

The study of the ABC transporters at the BAB is relatively recent and few studies have been performed, but it has been shown that P-gp and BCRP are expressed in the apical membrane of arachnoid barrier cells in humans, rats, and mice. MRP1 and MRP4 transcripts are detected in human BAB, and Mrp1, Mrp4, Mrp6, and Mrp7 proteins are expressed in rat (Table 2, Figure 2D))**.** P-gp (*ABCB1*, *Abcb1a* in mouse) and Bcrp (*Abcg2*) have been detected at the mRNA and protein levels in human, rat, mouse, pig, and monkey BAB cells, where they are located at the apical membrane [33,34,74,75,76,77,125]. MRP4 was also detected in pig and rat apical membranes by quantitative proteomics [34,125]. Importantly, other meningeal cells did not show the expression of these transporters in these studies. Moreover, Yasuda et al. [33] proved the functionality of P-gp at the AB, as they observed that its substrate daunomycin was accumulated in cultured mouse BAB cells after P-gp inhibition. Additionally, they showed the mRNA expression of *ABCC1* and *ABCC4* in human and mouse BAB cells, as well as other ADME-related genes (transporters and metabolism enzymes). More recently, Zhang et al. [34] used targeted MS proteomics to quantify several ABC and SLC transporters at the rat AB; obtaining the absolute expression of several ABC transporters (values in fmol/μg_protein_): P-gp/Mdr1a (16.6), Bcrp (3.27), Mrp1 (0.671), Mrp4 (0.510), Mrp6 (0.165), and Mrp7 (0.118), showing a high expression of P-gp and Bcrp, which was roughly twofold lower than in the rat BBB (24.9 and 6.95 fmol_monomer_/μg_protein_, respectively). Additionally, both studies showed the presence of other ADME-related genes’ mRNA transcripts and/or protein expression in the BAB cells, such as SLC transporters and metabolism enzymes. These results suggest that BAB could contribute importantly to the efflux of drugs from the brain, and thus it should not be neglected during drug delivery and clearance studies.

### 3.6. ABC Transporters Expression Differences between Animals

Care should be taken when translating results between different specifies and even animal strains or human populations because of differences in enzyme and transporters expression [93,126] (Table 2), and activity have been observed [127]. One important interspecies differences is MRP2/Mrp2 expression, which has been detected in rodents BMVEC (as mice and rats) but not in humans [128]. This can lead to the misinterpretation of pharmacokinetics studies of MRP2 substrates such as the anticancer drugs teniposide and etoposide [129], which are used clinically for recurrent the glioblastoma multiforme (GBM) treatment. In addition, absolute protein quantification using targeted tandem mass-spectrometry coupled to liquid chromatography (LC-MS/MS) has revealed different levels of ABC transporters in isolated brain microvessels in rodents and primates [93]. In rodents, P-gp has the highest expression among these proteins, followed by Bcrp and Mrp4. In primates, BCRP functional protein is slightly higher or at similar levels regarding P-gp and the expression of MRP4 is even smaller than in rodents. For instance, in the rat BBB, P-gp expression (24.9 fmol/μgprotein) is seven times higher than the functional homodimeric Bcrp (3.475 fmol_homodimer_/μgprotein) [93]. On the contrary, in human BBB, BCRP functional protein expression (3.07 fmol_homodimer_/μgprotein) is similar to P-gp (3.98 fmol/μgprotein) [22]. Interestingly, these studies showed that BCRP expression in humans was nearer to rodents than to other primates [93]. Importantly, these interspecies differences should be considered during drug development and specially when translating preclinical results to the design of clinical assays.

### 3.7. Conclusion on the Multidrug Resistance Related to ABC Transporters at the Blood–Brain Interfaces

ABC transporters are found in the three blood–brain interfaces to help maintain the brain homoeostasis. P-gp, BCRP, and the MRP family efflux diverse drugs, hindering their delivery for the treatment of CNS diseases. For a long time, research to study their function and overcome drug resistance has focused on the BBB, but recent works have located and shown their importance at the BCSFB and the BAB for drug clearance; therefore, they should not be neglected. Many models and methodologies have been useful to deepen our knowledge on the molecular and functional characterization of these barriers and to test new drugs and delivery strategies. Therefore, in the next section, we will summarize some of the most important methods related to the study of the localization, modulation, function, and implication in drug resistance of ABC transporters and the evaluation of drug penetration into the brain.

## 4. Methods to Study the Blood–Brain Interfaces

The development of new drugs or delivery strategies must pass through thorough evaluation in accordance to the regulatory agencies such as the American Food and Drug Administration (FDA) and the European Medicines Agency (EMA) [130,131]. This often includes testing for the physicochemical and pharmacological properties of drugs, such as lipophilicity and solubility, permeability prediction using Lipinski’s “rule of 5” [132], in vitro and ex vivo tests for pharmacological effect and toxicity of the molecule and its metabolites, in vivo analyses in animal models and eventually clinical trials. Particularly, in the case of CNS therapeutics, the pharmacokinetics of the drug or the effect of the delivery strategy should be assessed, considering its passage into the brain, and its clearance, through the blood–brain interfaces (the main pharmacokinetics calculations have been previously reviewed [5,133,134]). Therefore, many animal and human models have been developed for the study of the barriers’ phenotype, function, and changes, each one with its inherent advantages and disadvantages (further detailed in [4,5,6,7,8]). Some of the most used approaches to study the permeability and transport of drugs and to evaluate delivery strategies will be briefly described in this section.

### 4.1. In Vitro Models and Assays for Drug Evaluation

In vitro models mimic the barrier’s functional and/or anatomical characteristics with the advantage of being simpler and allowing the realization of experiments with higher control of the conditions and without the ethical concerns compared to in vivo assays. These models should present similar characteristics to the biological barriers, including the formation of a tightly closed monolayer, often measured as transendothelial electrical resistance (TEER) [135] or studying the permeability of hydrophilic tracer molecules such as Lucifer yellow, sodium fluorescein, sucrose, or mannitol. In addition, the cells should express the correspondent proteins at specific subcellular localization (polarized), including tight junctions, transporters, enzymes, signaling receptors and pathways, as well as macromolecular and immune cell trafficking [7,136,137]. In vitro assays are required for the screening and validation of new drugs; they are used to evaluate cytotoxicity, metabolism-mediated interactions, transporter-mediated interactions, and drug–drug interactions according to drug regulatory agencies such as the FDA and EMA [130,131] In the case of CNS drug delivery, they are useful to address BBB permeability and evaluate delivery strategies [5,7].

**Figure 3:**
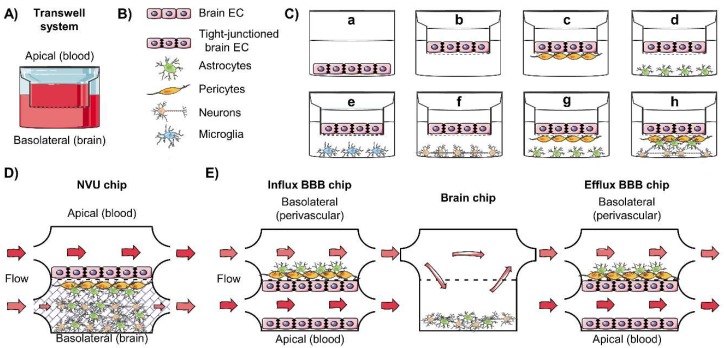
Schemas of examples of in vitro models of the blood–brain barrier (BBB)**.** (**A**) Cells conforming the neurovascular unit (NVU) used for cell culture, usually in (**B**) Transwell systems with an apical (blood) and basolateral (brain) space, separated by a permeable membrane (dashed line). (**C**) Primary or cell lines of brain endothelial cells (EC) can be cultured (**a**) directly in wells to study drug absorption or (**b**) in transwells for permeability assays; (**c** to **f**) cocultures with other cells from the NVU can improve the BBB phenotype, including the tight junctions (black rhombus ♦); (**g** and **h**). Furthermore, a synergic effect is observed when several cell types are cocultured. (**D**) and (**E**) are examples of BBB-on-chip systems. (**D**) Two-chamber microfluidics system developed by Brown et al. [188]. (**E**) Multichamber system developed by Maoz et al. [189] consisting of a brain chip connected to an influx BBB chip and an efflux BBB chip, which allowed the study of drug BBB permeability and clearance. Created using images from “smart Servier Medical Art”, Creative Commons License, 2019.

Permeability studies can be performed using devices consisting of a permeable filter separating an apical (luminal) and a basal (abluminal) compartments such as the Transwell removable inserts (Figure 3A). The endothelial cells are grown to confluence on the filter to form a tight monolayer which should obtain a polarized phenotype as similar as possible to the in vivo, including specifically located transporters and high tightness [7,136,137]. Two different mediums can be used to mimic the apical space on top of the filter and the basolateral below. After adding the testing molecule to the acceptor compartment, samples are taken overtime from the donor medium. The incremental clearance volume (ΔV_Cl_) on each time is calculated as the product of the concentration in acceptor (C_a_) by its volume (V_a_), which is divided by the concentration in the donor (C_d_) (ΔV_Cl_ = [C_a_ * V_a_]/C_d_); the slope of the linear curve is divided by the surface of the filter to obtain the total permeability, which is corrected to account for cell-free areas of the filter. Normally, the permeability from the apical to basal compartment is assessed, but the opposite sense can be used to evaluate efflux transport [133,134].

Brain endothelial cell uptake can be evaluated by incubating the cell monolayer (Figure 3C) with a tracer molecule. The uptake process is quenched at serial time points using a cold buffer solution or adding transport inhibitors; then, the cells are lysed, and the total proteins and tracer concentrations are measured using scintillation (for radio-labeled compounds) or other methods such as liquid chromatography (LC) coupled to UV-visible detection (LC-UV) or to mass spectrometry (LC-MS), for instance. The volume of distribution of the test substance (V_d_, in μL mg^−1^_protein_) is calculated as the ratio of counts or amount of the substance per milligram of proteins to the ratio of counts or amount of the substance per microliter of incubation medium. If the uptake is mediated by transporters, Michaelis–Menten kinetics can be obtained using nonlinear regression analysis of the concentration dependence of the influx [133].

Diverse strategies can be employed to evaluate the implication of ABC transporters in the drug efflux [5,133,134]. Inhibitors of the ABC transporters specifically targeting one or more of them can be employed to evaluate their implication in the substance permeability or efflux by comparing with the uninhibited condition. For instance, tariquidar and elacridar can inhibit both P-gp and BCRP, while verapamil, N-desmethyl-loperamide, and loperamide can target P-gp specifically (detailed in Section 6.1). Similarly, the transporter expression can be knocked-out or knocked-down, or models overexpressing an ABC transporter can be compared [133]. Endothelial cell models can be used as a surrogate of the blood–brain interfaces to study the ABC-related transport [5], such as the human colonic epithelial cell line (Caco-2) or the Madin–Darby canine kidney cell line (MDCK) epithelial cells used to predict the gastrointestinal permeability of a compound or drug. These have the advantage of being simple, widely used, and MDCK having been engineered to specifically overexpress ABC transporters, such as P-gp [138]. Nonetheless, results should be interpreted carefully, as these cells do not represent exactly the mechanisms driving brain permeability due to considerably lower tightness and differences in the expression of ABC transporters [133]. Therefore, there are continuous efforts to develop in vitro models of the blood–brain interfaces to improve the evaluation of drug delivery into the CNS, which will be briefly summarized in the following subsections, as their advantages, disadvantages and molecular characterization have been thoroughly reviewed recently [4,7,136,139,140,141,142,143]. Importantly, for a wide review on the receptor and transporter expression in diverse in vitro models of the BBB, including data on ABC transporters, please refer to [7].

### 4.2. In Vitro Models of the BBB

Drug permeation is often studied using models of the BBB, which is considered as the main interface for brain delivery into the brain. These cell models are generated from primary cultures or immortalized cell lines of brain capillary endothelial cells [4] from mouse [144], rat [145,146,147,148,149], bovine [150], porcine [151], Rhesus macaque [152] and human [153,154,155] models, and more recently from endothelial cells derived from human stem cells [156,157,158,159] (reviewed in [6,7,136,160]). Primary cultures of endothelial cells are obtained by culturing brain microvessels obtained by an enzymatic dissociation and cultured with specialized media to eliminate astrocytes and pericytes [147,149,155,161]. Endothelial cells can be immortalized using different strategies (e.g., the E1A adenovirous gene, [162]) and have the advantage that they can be used repeatedly and shared between laboratories. The human immortalized endothelial cell line hCMEC/D3 [153,154] is widely used for the study of drug transport [163,164,165,166] because it has been thoroughly characterized and it expresses ABC and SLC transporters, as well as tight junctions [167,168,169], despite the fact that the tightness of its monolayer is lower than in intact microvessels due to a lower expression of claudin-5 [169]. Recently, human brain endothelial cells have been obtained from stem cells such as human cord blood-derived stem cells of circulating endothelial progenitor and hematopoietic lineages [156,157], human pluripotent stem cells (hPSCs) [158], and induced pluripotent stem cells (iPSCs) [170,171]. After differentiation and isolation, the hPSC-derived brain endothelial cells monolayers present key BBB characteristics, including tight junctions and functional ABC transporters [158,159,172].

Several strategies have been employed to improve the BBB functions of cultured endothelial cells. As mentioned in Section 2.1, the BMVEC phenotype and the BBB function depend on their dynamic interactions with the NVU (Figure 3B); therefore, monocultures of endothelial cells may present lower tightness (Figure 3C), different ABC transporters expression and higher permeability [6,7,136,160]. The indirect interactions have been studied in vitro and they have been exploited to improve the tightness and functional expression of transporters in BBB models [4,7]. Diverse studies have employed, for instance, soluble factors such as retinoic acid, cAMP, cytokines, growth factors, and neurotrophic factors [147,159,173]; astrocyte-, pericyte-, or neuron-conditioned media [82,173,174,175]; or glial-derived extracellular matrix [175]. An improvement in the expression of tight junctions and BBB phenotypes have been observed when brain endothelial cells are cocultured in two-chamber systems with astrocytes [144,149,176,177,178], pericytes [179], neurons [180], or microglia [181]; furthermore, a synergic effect is seen when several cell types of the NVU are cocultured (Figure 3C) [6,7,136,160,182]. The different cells can be placed in direct or indirect contact in these models, but this can also impact the BBB phenotype [182]. Therefore, the nearest to an in vivo model should be used, but this can be time and resource consuming; thus, simple models can also be useful for drug screening, depending on the objective of the experiment.

Cocultures have been widely employed in the recent years for the evaluation of drug permeability. A commonly used approach to better mimic the BBB consists on the coculture of a rat primary culture [183] or cell line of astrocytes [184] with BMVEC from rat [147,149,185], porcine [183,184], bovine [177,178,186] or human cells to obtain an improved barrier phenotype characterized by a higher TEER or even increased functionality of ABC transporters [186], as observed for P-gp in a rat astrocyte–bovine brain endothelial cells system [187]. For instance, the tightness of the monolayer formed by hPSC-derived brain endothelial cells is increased by coculture with rat astrocytes [158]. In addition, some enterprises commercialize human primary cells of astrocytes, pericytes, BMVEC, and neurons that can be used for in vitro modeling of the BBB, which recently allowed the evaluation and comparison of complex cocultures containing combinations of these components of the NVU [182].

### 4.3. Dynamic In Vitro Model, Toward the BBB-on-Chip

The BBB phenotype depends on the interaction of the endothelial and glial cells, but the hemodynamic forces, such as the shear stress and cyclic strain, are also a key factor modulating vascular endothelial cells [190], which has been exploited to improve BBB models. When endothelial cells are cultured under a laminar flow, their morphology is more similar to the in vivo than in the absence of flow [142,191,192,193]. The frictions forces applied by the flow at the apical surface of the endothelium activate diverse mechanosensors such as caveolae, ion channels, PECAM-1, integrins, cadherins, G proteins, and kinases, which are involved in the signaling pathways that regulate cell differentiation [194,195,196,197,198]. This results in larger and flattened endothelial cells, with improved tightness (higher TEER), increased gene and protein expression of primary metabolism pathways, adhesion and tight junctions, CYPs450, ABC transporters, and ion channels [142,196,197,199]. Importantly, flow contributes to the polarization of the endothelial cells, including the localized (apical or basolateral) expression of functional transport systems such as ABC and SLC transporters and endocytosis mechanisms [142,200,201,202]. Diverse devices have been employed to study the effects of shear forces on the endothelial cells phenotype, starting with the use of a viscosimeter adapted with a cone plate to induce the fluid shear stress in culture plates [198,203]. However, the need for a precise control of the laminal flow and of coculturing the endothelial cells with glial cells to improve the BBB phenotype has pushed forward the development of more performant and more complex “BBB-on-chip” models.

BBB-on-chip models created using 3D devices allow a better representation of the barrier function by including a laminal flow of culture medium to mimic the blood stream and often coculturing endothelial and glial cells (reviewed in [139,141]). The team of professor Janigro pioneered the development of BBB coculture models under flux in the late 1990s, allowing for the first time the real time measurement of permeability for dynamic studies [200,202,204,205]. They developed a tridimensional device consisting of a hollow-fiber tube with medium flowing inside it, where endothelial cells can be cocultured with astrocytes, obtaining an improved formation of tight junctions, a resistivity nearer to in vivo conditions, the polarized expression of transporters, and selective permeability [202]. Diverse systems have been assessed, using mono and cocultures of animals, human, cell lines or mixed-origin cells and different devices. Although none of these was perfect, the 3D models are nearer to the in vivo assays, while conserving the advantages of controlled conditions of in vitro assays. Furthermore, using differentiated human iPSC cells, these in vitro systems can better mimic the human BBB and its changes in disease by using patient cells, which could even lead to the development precision medicine strategies (reviewed in [139]).

Recent advances in microfluidics (reviewed in [206]) and cell culture have allowed the creation of very complex models of the “NVU-on-chip”. Brown et al. [188] developed a device consisting of a two-chamber system separated by a porous membrane, which is covered at the blood side with a monolayer of endothelial cells and with pericytes and astrocytes at the side of the brain chamber, filled with iPSC-derived human cortical neurons and codifferentiated astrocytes fixed in a collagen gel [188] (Figure 3D), allowing the dynamic study of the BBB response to inflammation [207]. Maoz et al. [189] modeled not only brain permeability but also the clearance through the BBB by connecting a brain chip between the perivascular spaces of two BBB chips (influx and efflux) (Figure 3D). The BBB compartments consisted of a monolayer of human brain microvascular endothelial cells at the vascular chamber and pericytes and astrocytes at the perivascular chamber, while the brain compartment contained neurons and astrocytes at the lower chamber. This multichamber system allowed the study of the individual contribution of the NVU cells to the maintain of brain functions through metabolic interactions [189]. More recently, the same team developed a single two-chamber chip improved model using pluripotent stem cell-derived human brain microvascular endothelium interfaced with primary human brain astrocytes and pericytes (but not neurons) and including a period of differentiation under hypoxic conditions using a “developmentally-inspired induction protocol”, which resulted in an increase in the expression of ABC and SLC transporters compared to normoxic conditions [208].

### 4.4. In Vitro Models of the BCSFB and BAB

There has been a lower interest in the development, use, and characterization of in vitro models of the BCSFB and BAB, but they should not be neglected, considering their barrier function and their importance for drug delivery into the CNS and its clearance, as mentioned in Section 3. Nevertheless, the new discoveries on their implication in drug delivery and clearance may push forward to the development of more complex and better models. Furthermore, interconnected multichamber microfluidics systems, similarly to the one employed by Maoz et al. to study the BBB [189] (Figure 3D), could be employed to create a “brain-on-chip” system including all the three blood–brain interfaces and used to improve our understanding of brain pharmacokinetics.

Several in vitro models of the CP have been developed to study the BCSFB function [209], including mouse [210], rat [211], porcine [212,213] and Rhesus macaque [214] primary epithelial cells and immortalized mouse and rat cells [209,211,215,216]. These models can be used to study drug delivery and clearance, as well as transporter function; for instance, the porcine model of CP epithelial cells developed by Baeh et al. [213] formed a monolayer with key characteristics of the BCSFB; such as the expression of Mrp1 in the basolateral (blood-facing) membrane, similarly to its location in tissue.

Cultured primary or immortalized arachnoid cells have been used as in vitro models of the BAB [217]. These models have been of great importance for the discovery of their barrier function; this includes the discovery of ABC and SLC transporters in immortalized cultures of mouse BAB cells [33], the study of junctional proteins in a human primary line obtained from arachnoid granulations [218], and its interaction with blood in immortalized [217] and primary rat arachnoid cells [219].

### 4.5. Ex Vivo Models

Ex vivo models try to represent the architecture of the blood–brain interfaces better than in in vitro assays, while allowing a faster and easier evaluation of drug transport or distribution than in in vivo models. The living tissue is extracted from the organism and placed in an artificial environment, taking care of minimizing the disruption of the sample to achieve a maximum similarity to in vivo conditions. This allows performing studies in very controlled conditions that would be impossible in living specimens [4]. Tissue slices are advantageous as they conserve the cytoarchitecture of the tissue, maintaining thus the interactions between the cells and miming the brain environment. They can be used to study the bound and unbound amount of a compound incubated with the brain slice, allowing the study of transporter function [220]. In addition, immunohistochemistry (IHC) studies, in situ hybridation, mass-spectrometry imaging, or other studies can be performed to study molecular pathways and their modulation after compound administration for drug development [4,221].

Diverse strategies have been devised for the ex vivo study of the brain barriers separately. Isolated microvessels can be employed for the molecular characterization of the BBB and NVU [21,22,95,168,222], used immediately for functionality studies [223,224,225,226,227], or cultured with specific media for a limited time [149]. Whole brains or specific sections (e.g., cortex) are homogenized either mechanically, employing a Potter–Evenheilm homogenizer [115,161,228] by enzymatic dissociation with collagenases that partly degrade the basement membrane [146] or using both [229]; then, the microvessels (diameter <10 μm) are separated from other cells using a density gradient and isolated from bigger vessels by sequential filtration on nylon meshes. Choroid plexus explants can be obtained by carefully dissecting the choroid plexus from the brain ventricles and immediately placing it in specialized media for functional studies of the BCSFB [230]. For instance, this has been employed to evaluate peptides for ligand-mediated targeting to CP epithelial cells as a strategy for CNS drug delivery [231,232]. Similarly, the BAB can be studied using meninges explants by carefully dissecting the meningeal layer, then separating the leptomeninges containing the arachnoid cells from the dura mater [34]. Furthermore, permeability assays can be performed using the whole meningeal layer in a diffusion chamber system [233] or a perfusion system [234].

### 4.6. In Vivo Models and Assays

In vivo assays are necessary to study drug delivery to the brain. Preclinical tests using animal models are necessary to study the pharmacokinetics and pharmacodynamics of new drugs and delivery strategies, as well as the processes driving their ADME, as required by drug validation agencies [130]. In the case of CNS therapeutics, the entry into the brain and its clearance are key factors for drug efficacy, and their ADME is governed by complex mechanisms that are not perfectly mimicked by in vitro or ex vivo models; in addition to that, other organs also interfere in their ADME [5,6,8]. The methods and conditions that can be used for the study of animal specimens are limited due to ethical and practical considerations, as well as due to the equipment availability and prices; therefore, in vivo experiments should be carefully planned, complying with the local regulations [130,131].

Many animal models and techniques have been developed to obtain valuable information about drug ADME at the brain. Mouse and rat models are often used to characterize the brain barriers and study CNS drug delivery and ADME, but dog and non-human primates such as the as monkey and Rhesus macaque have also been employed. Similarly to in in vitro assays, inhibitors of the ABC transporters can be employed to evaluate their implication in the substance permeability or efflux by comparing with the uninhibited condition [5]. In addition, knock-out and knock-down models of one or more of the ABC transporters have been developed to evaluate their implication in the permeability and efflux of testing molecules [5,235]. It is important to remember that there can be molecular and physiological differences between species, such as expression levels, substrate specificity, and transport efficiency in the case of ABC transporters (see Section 3.6). Therefore, models with humanized ABC transporters, as P-gp and BCRP, have been developed to better represent drug transport in humans [236,237,238,239]. Diverse techniques are used to evaluate the permeability of molecules into the brain and their clearance and obtain important ADME, toxicity, and PK-PD parameters (reviewed in [5,6,8,133,240]. Some of the most important will be summarized below.

In intravenous (IV) infusion, a labeled or unlabeled version of the studied compound is injected or infused (to maintain a steady level in plasma) and the concentration in plasma across the time and in a terminal brain sample is measured using, for instance, scintillation, LC-UV, or LC-MS, as well as imaging techniques (Section 4.7). The concentration versus time plot in plasma is used to calculate the area under the curve (AUC), and the concentration in the brain is corrected for residual intravascular tracer to estimate the amount entering the brain parenchyma [6].

In situ brain perfusion (ISBP) is an invasive method used on animals for the study of the compound’s permeability through the BBB [241]. In this method, the animals are infused in a time and flow-controlled manner with a perfusate (bicarbonate buffer, plasma, or blood) via the carotid artery. The flow rate and composition of the perfusate can be modulated to study different kinetic parameters such as blood and brain concentration (i.e., permeability and clearance) or transport modulation [227,242,243,244].

### 4.7. Imaging Methods

Imaging methods allow the evaluation not only of pharmacokinetics parameters, but also of the specific distribution of drugs and their metabolites. Although they are mostly used for in vivo analyses, they can also be employed to study in vitro models. This includes nuclear imaging techniques that are non-invasive or minimally invasive such as positron emission tomography (PET), single photon emission computed tomography (SPECT), and magnetic resonance imaging (MRI); but also mass spectrometry (MS)-based imaging (MSI) that although is a destructive technique, its multiplexing capacity and spatial resolution can be exploited for preclinical studies. The nuclear imaging techniques for the study drug transporter function have been thoroughly reviewed recently [245]; thus, in this section, we will only address PET as an example, and then summarize MSI application to the study of drug delivery.

Positron emission tomography (PET) is a functional imaging technique used to study drug distribution in the body and the CNS and is increasingly being used in all stages of CNS drug development (reviewed in [245,246,247,248]). A molecule labeled with short half-life radioactive isotopes is administrated to an animal or human, and the positrons emitted by this tracer are detected and interpreted by the PET detector, allowing the study of compound’s spatial distribution and its quantification in function of time [245,247]. Although PET depends on the availability or production of the radiolabeled analyte, it has the advantages of being a non-invasive technique that can provide dynamic data for in vitro, animal in vivo or human clinical analyses.

Radiolabeled substrates or inhibitors of the ABC transporters can be used to study their function, but one of the difficulties encountered is the need for specific probes due to overlapping substrate and inhibitor affinities (Section 3.2). [^11^C]erlotinib [249,250,251], [^11^C]elacridar [252,253,254,255], [^11^C]tariquidar [253,254,256,257], and [^11^C]temozolomide [258] have been used to trace the concomitant function of P-gp and BCRP in the brain. [^11^C]verapamil [259,260,261,262], [^11^C]loperamide, and [^11^C]N-desmethyl-loperamide [263,264,265] have been used to specifically visualize P-gp function because they are not transported by BCRP. To date, there is no specific PET tracer for BCRP, as the compounds developed as specific molecules, such as [^67^Ga]Galmydar, showed no significant difference between BCRP knockout and wild-type mice or rats [266,267,268], and it would not be ideal to substrate the specific activity of P-gp from that of a P-gp/BCRP common substrate, as their function is synergic and not additive [53,98]. Thus, other strategies have been proposed, such as the use of the P-gp/BCRP substrate [^11^C]tariquidar, which was coadministered with unlabeled tariquidar to inhibit P-gp at the BBB [256]. 6-bromo-7-[^11^C]methylpurine has been employed to specifically visualize MRP1 function in the brain, as it is transformed to its glutathione conjugate after passively crossing the BBB and then effluxed by MRP1 [269].

Among other applications, TEP has been used to asses P-gp and BCRP function and inhibition in mouse [238,253,270], rat [261,271], primate [250,264] and human [251,254] models; and it could be used for precision medicine through the evaluation of individual variability in response to CNS drugs [272]. In addition, diverse strategies have been developed to study for instance drug–drug interactions of unlabeled molecules in combination with already available tracers [246]. PET was recently employed to validate the permeability and transporter function of a human iPSCs BBB model [172] and the evaluation of ABC transporter-humanized mice models [238,270]. Importantly, it has been used to evaluate drug delivery strategies, such as inhibition [250,251] or focused ultrasounds [273] (see Section 6.5 for details).

Mass spectrometry-based imaging (MSI) can be used to assess the localization of small and large molecules in tissues and in vitro samples. Employing a mass spectrometer, molecules are desorbed from the sample and ionized using one of many techniques such as matrix-assisted laser desorption (MALDI) or desorption electrospray ionization (DESI). The ions are detected by MS in a position-specific manner, which allows the reconstruction of the distribution of one or hundreds of molecules, such as metabolites, lipids, peptides, or proteins, over the sample’s surface. MSI has the advantage of allowing the location and even quantification of a large diversity of molecules in a single assay, including drugs and their metabolites, without the need for the labeled analyte, although the spiking of heavy stable isotope standards is preferred for quantification. Nevertheless, it is a destructive method that cannot be used for true in vivo imaging and needs method optimization depending on the characteristics of the searched analytes [274,275].

MSI can be used for the molecular characterization of proteins and small molecules of tissues or tumors [276,277,278], drug distribution, and BBB permeability [279] for pharmacokinetics and pharmacodynamics analyses [280] and is becoming a powerful tool for drug development. MALDI-MSI has been used to visualize drug penetration in brain tissue in an elegant strategy where the hemoglobin cofactor heme was used as a marker of brain and glioma vasculature [279]. They showed that RAF265 (CHIR-265), a small molecule inhibitor of the RAF serine/threonine protein kinases’ permeability into the brain, is limited by the BBB. Using a spatial resolution of 25 μm, they were able to determine that RAF265 accumulated within the vascular lumen of intracranial tumor implants in mice, but did not cross the BBTB, which could not be resolved using PET. Both MALDI-MSI and DESI-MSI have been suggested as powerful tools for the rapid molecular diagnosis of human brain tumors by studying lipid [281,282,283] or protein signatures [284] in tumor biopsies; in addition, DESI-MSI has the advantage of needing minimal to null sample treatment, which could be used for intraoperative diagnosis and exploited for the development of precision medicine strategies [280].

### 4.8. In Silico Models

In silico models can be used to computationally predict the ADME of compounds during CNS drug development. Using mathematical modeling, the permeability of a molecule across the BBB, its distribution in the brain, the binding to its target and/or its clearance can be predicted based on the compound’s physicochemical properties, allowing the screening of thousands of drug candidates using computer calculations [6,132]. Although it still needed to perform some experiments to feed the models or to confirm their results, this is considerably diminished, reducing this time and money-consuming step of drug development to fewer molecules with better pharmacokinetic and pharmacodynamic characteristics. In addition, there is a continuous work in developing more accurate and faster algorithms as tools for drug screening and many exist today, as has been reviewed previously [4,6,285]; thus, some of the most important will be explained below.

Some models are based on physicochemical properties as the lipophilicity [286] or quantitative structure–property relationships (QSPR) [287] to predict the BBB permeability and brain distribution of compounds [285]. Artificial intelligence (AI) models [288], such as machine learning, have been employed to predict the BBB permeability represented as unbound brain to a plasma concentration ratio of small molecules [289] or even peptides [290]. Machine learning uses a training dataset with known parameters to predict those parameters of an unknown dataset employing linear regression or more complex algorithms such as artificial neural networks. For instance, the QSPRs of diverse molecules with known physicochemical properties and permeability can be used to predict the permeability of a larger group of new untested molecules [289,290].

In physiologically based pharmacokinetic (PBPK) models, in silico predictions or experimental data from in vitro or in vivo studies are used as input parameters in an algorithm to predict the time-dependent distribution of the molecule between compartments (fluid chambers, tissues, or groups of tissues with similar characteristics) [291,292]. This compartment-based approach allows taking into account physiologically meaningful parameters such as tissue volumes and blood flows in addition to drug-specific biochemical parameters such as the transport mechanisms and enzymatic metabolism [293,294].

In in vitro to in vivo extrapolation (IVIVE), in vivo drug transport, or metabolism is predicted from data obtained in vitro, using correction parameters such as the relative expression factor (REF) and relative activity factors (RAF). This allows a reduction of the number of assays using animals, by employing data acquired with cultured cells [126,295,296].

### 4.9. Recent Molecular Characterization Techniques

Although the mechanisms underlaying the brain barriers phenotypes and function have not been completely elucidated, there has been continuous work for their molecular characterization in health disease and in response to drugs, using a broad range of methods. Classical technologies have been largely used for this purpose, including RT-qPCR, Western blot, IHC, and in situ hybridization. Nevertheless, the advent of new “omics” technologies has allowed a broader and deeper understanding of the blood–brain interfaces and their role in drug delivery; including diverse transcriptomics and proteomics of brain microvessels, as well as cell-specific studies [64,65,85,297,298,299,300]. In addition, in vitro models and NVU-on-chip systems have been characterized using transcriptomics, proteomics, and metabolomics allowing not only the validation of the models, but also improving our knowledge on the interactions between brain cells [22,188,189,207,208]. Large-scale programs as the Human Protein Atlas [68] are also an important tool for the study of protein expression in the brain, cancer, and cultured cells. Altogether, new and classic technologies help the scientific community to obtain a broader understanding of brain cancer and its drug resistance in order to develop better treatment and drug delivery strategies.

Proteomics is a powerful tool used to study dynamic protein expression and their regulation, mainly using MS. Diverse strategies have been used for this purpose, as the work performed by the team of Professor Karamanos using 2D-gel electrophoresis and MS protein identification (reviewed in [301]). Nevertheless, membrane proteins such as ABC transporters are difficult to detect by this method due to their physicochemical properties and low abundance. Targeted liquid chromatography coupled to tandem mass spectrometry (LC-MS/MS) analysis of digested proteins gives the sensitivity need for the quantification of ABC transporters, SLCs, and CYPs450 [94,297,302,303]. Furthermore, using stable-isotope labeled standards [304], protein abundance can be reported in non-arbitrary units as fmol of molecule per μg of total protein with high accuracy, precision, and selectivity [303]. The results of this absolute quantification can be used to study transporter expression modulation due to diseases or drug exposure [223], interspecies [93], or even human interindividual variability [305]; besides, results have been used to calculate the relative expression factors (REF) for IVIVE [126,295] and PBPK modeling (see [306] for recommendations). In addition, the specificity of these methods has been exploited to characterize and validate murine models with humanized ABC transporters [236,237,238,270].

### 4.10. Conclusion on Methods to Study the Blood–Brain Interfaces

Diverse in vitro, in vivo and in silico models have helped us to study the implication of ABC transporters in drug resistance, and they are needed for the development and validation of new drugs and delivery strategies to treat CNS diseases. Gliomas are brain cancers that represent a particular challenge as they are highly disabling for the patients and are often recursive, causing high mortality rates [9,10]. This is partly due to the multidrug resistance of cancerous cells and the difficulty of delivering chemotherapeutics to the brain, both being highly related with the efflux by ABC transporters, as will be explained in the next section.

## 5. Implication of ABC Transporters in the Multidrug Resistance of Glioma

Cancers of the central nervous system (CNS), and particularly gliomas, represent a worldwide problem for healthcare because of its high morbidity and mortality. Patients of CNS cancers become highly disabled by the disease, treatments are expensive, and prognosis is low, due to the tumor’s aggressiveness and resistance to multiple chemotherapeutic drugs [9,10]. Importantly, the blood–brain tumor barrier (BBTB) can be disrupted in glioblastomas, but glioma cells can invade zones with intact barrier function [307]. Additionally, lower grade gliomas can present a BBTB similar to the BBB and even an increased barrier function, hindering the entry of therapeutics [14,16]. In this section, we will briefly introduce the pathology of glioma and summarize the implication of the blood–brain interfaces in drug resistance, particularly the role of ABC transporters, and some strategies to study and improve anticancer drug transport and delivery.

### 5.1. Glioma Classification

Gliomas are brain tumors originated from astrocytes, oligodendrocytes, or ependymal cells. According to the World Health Organization (WHO) Classification of Tumors of the Nervous System, gliomas are classified by their histology and molecular features, in addition to their grade of malignancy (from grade I to grade IV) [10,11]. Grade I gliomas are benign tumors; the cells look almost normal in microscopy, they grow slowly, they are compartmentalized, and surgery alone may be enough for their treatment. Grade II (low grade diffuse glioma) tumors are slow growing, with an abnormal phenotype, and some can diffuse to nearby normal tissue, which can lead to recurrence after surgery. Grade III (malignant/anaplastic diffuse glioma) tumors are malignant, the cells reproduce at abnormal rates and diffuse into nearby normal brain tissue; they can evolve into grade IV tumors. Grade IV tumors present abnormal cells, reproduce rapidly, may be resistant to apoptosis; these often diffuse into the surrounding normal brain tissue and have angiogenic capacities to maintain the blood supply and support the rapid growth. They also may present necrotic zones in the interior. The most common example of a grade IV tumor is the glioblastoma multiforme (GBM) that is characterized by a very low rate/time of survival [11]. GBMs are subdivided in primary GBM, which are originated de novo, and secondary GBM, which displays evidence of progression from a lower-grade tumor. Primary GBMs are more frequent, representing around 95% of the diagnosed cases [9,308].

Gliomas are subclassified in diverse groups with different histologic and molecular characteristics. Historically, gliomas have been grouped mainly by histologic studies [11], but the most recent WHO classification [10,13] includes molecular diagnostic criteria that can be important for treatment and prognosis, such as the analysis of isocitrate dehydrogenase (IDH) mutation, histone mutations, and chromosome 1p/19q deletion. For instance, (IDH) mutation generates proteins that metabolize α-ketoglutarate into 2-hydroxyglutarate (2HG), which is a possible oncometabolite [309]. Mutated IDH has been detected in many low-grade gliomas (mainly astrocytomas and oligodendrogliomas) and secondary GBMs, but only in some primary GBMs [13,310,311], and it has been related with a better progression-free survival than IDH wild-type gliomas [312]. This has driven to the development of methods to diagnose *IDH* mutation status before biopsy using magnetic resonance spectroscopy [313] and strategies to target IDH using inhibitors and targeted vaccines [314]. Thus, it is important to consider genomic, epigenomic, and proteomic markers for the classification and subclassification of gliomas, not only for the diagnostic, but also for the treatment and prognosis of patients.

### 5.2. Epidemiology and Prognosis

Gliomas are the prevailing category of CNS cancers, whose incidence rate has increased in the last decades [9,315]. Gliomas represent more than 77% of the brain neoplasms diagnosed globally [315,316], and according to the Global Burden of Disease Study, 330,000 (with 95% uncertainty intervals [95% UI] 299,000 to 349,000) new cases of primary malignant brain tumors were diagnosed in 2016. This study reported a significant increase of 17.3% in the global age-standardized incidence rate between 1990 and 2016 [9], growing in almost all geographical regions (except for eastern Europe) and Socio-demographic Index (SDI) quintiles.

The prognosis of glioma patients depends on several factors, including the grade and subtype, age at diagnosis, extent of tumor resection, and the Karnofsky performance status (KPS) [316,317,318]. The five-year survival rate in several regions including US, Korea, and Europe has been reviewed previously by Ostrom et al. (2014) [316]. In general, gliomas with the oligodendroglial phenotype have a greater survival rate than astrocytic gliomas. Grade I astrocytomas (pilocytic astrocytomas) can be treated by surgical resection and have the highest rate of survival. Prognosis for grade II gliomas is relatively good, with 47.8% to 79.1% of the oligodendrogliomas patients surviving for five years and 28% to 51.6% surviving for five years in the case of astrocytomas. Nevertheless, grade II gliomas can be infiltrative, become more malignant due to genetic alterations, and evolve to grade III (anaplastic gliomas) [318]. Grade III gliomas present a reduced prognosis, with a five-year survival inferior to 50% for anaplastic oligodendrogliomas and inferior to 30% for anaplastic astrocytoma. GBM is the most aggressive type and less than 5% of patients survive for more than five years after diagnosis, despite the implementation of different therapies (e.g., chemotherapy, irradiation, etc.) [316,317,319].

### 5.3. Multidrug Resistance in Glioma and the Blood–Brain Tumor Barrier (BBTB)

Gliomas are normally treated by surgically extracting the tumor as full as possible, which is difficult for infiltrating tumors, followed by radiotherapy. Systemic chemotherapy can be used for patients with progressive disease or high grade gliomas (e.g., GBM), but its efficacy is often poor because most of the brain tumors are resistant to multiple structurally unrelated classes of anticancer drugs [12,14,16]. Thus, none of the treatments currently available is curative, and the prognosis in high grade gliomas is very low [316,317,319].

The mechanisms of multidrug resistance in cancer cells are multifactorial and have not been completely elucidated. The tumors’ drug resistance can be improved due to genetic and epigenetic alterations, the up- or down-regulation of genes, and changes in the post-translational regulation of protein activity [12,14,16,320]. These mechanisms can include an increased drug efflux due to the presence and possible up-regulation of drug efflux transporters such as ABC transporters [16] and some members of the SLC superfamily [14,108,121]. In addition, a reduced uptake without increased efflux has also been observed, as in the case of the antifolate methotrexate [321]. Detoxifying systems such as drug metabolizing enzymes such as CYPs450 can convert the anticancer drug to less toxic metabolites [309,322]. The down-regulation of apoptotic pathways, either as a result of the malignant transformation (e.g., p53 mutations [323]) or during exposure to chemotherapy such as the alteration of ceramide levels [324]; additionally, modifications in the cell-cycle machinery or DNA repairing mechanisms can prevent apoptosis [325]. This multidrug resistance leads to poor treatment efficiency, cancer relapse, and eventually death [16,316,317,326]. In addition to these mechanisms, drug distribution to brain tumors is often hampered by the natural barriers protecting the brain [14].

The blood–brain tumor barrier (BBTB) is formed by vascular capillaries with altered barrier function. Each tumor case is different, presenting its own morphological and physiological characteristics both in the cancer and endothelial cells. Normally, low-grade gliomas show a normal vascularization where the BBB remains mostly intact, although some molecular changes can occur, such as transporter expression modulation [327]. High-grade gliomas present more alterations, including an increased vascularization [307,328] with heterogeneous undisrupted and disrupted leaky zones that allow the exchange of big molecules as proteins and even cells, which can lead to cancer metastasis [329]. The levels of ABC transporters normally expressed at the BBB can be up-regulated at the BBTB, and ABC transporters can also be expressed in non-vascular tumor cells, improving their defense against chemotherapeutics (Section 5.4) [14,16,108,121,325,326]. In summary, both the BBB and the BBTB represent major obstacles for anticancer drug delivery in low and high-grade glioma [14].

The BBTB is heterogeneous and can present leaky zones but still forms a barrier against drug penetration into the tumor. It is often assumed that the BBTB is completely disrupted; thus, its barrier function is often neglected during GBM drug design [329,330], but there are increasing clinical evidences showing that all the GBM present tumor zones with intact BBB, hindering drug permeability into the tumor (reviewed in [330]). This is supported by diverse murine studies showing a low drug penetration into tumors of xenografted mice [331,332,333] and PDGF-B–driven brainstem glioma models [334], due to ABC transporters P-gp and Bcrp [333,334]. In addition, a recent study using 3D-MSI showed a higher but non-homogeneous accumulation of erlotinib in the GBM tumors of xenografted mice than in brain parenchyma, proving that the BBTB is heterogeneously disrupted across the tumor [335].

### 5.4. ABC Transporters Role in Glioma Drug Resistance

The expression of ABC transporters in cancer cells and the BBB/BBTB has been directly related to chemoresistance against several of their anticancer drug substrates [42,52,53,56,336]. The presence of ABC transporters in both the BBTB and the tumor cancer cells indicate a multibarrier system defending the cancer cells from chemotherapeutics, coupled to detoxifying systems and the anti-apoptotic machinery. *ABCB1*/P-gp, *ABCG2*/BCRP, *ABCC1*/MRP1, ABCC4/MRP4, and *ABCC5*/MRP5 [14,16,104] up-regulation has been observed in glioma cells at the mRNA and/or protein level (Table 3); moreover, MRP3 de novo protein expression has also been detected in high grade gliomas (detailed below) [16,337]. This can lead to resistance to their multiple chemotherapeutic substrates (Table 2), as it has been proven for doxorubicin, vincristine, etoposide, camptothecin, topotecan, methotrexate, sunitinib, imatinib mesylate (Gleevec), and palbociclib [16,104,106,108,333,338,339,340]. Furthermore, their expression is heterogenous even between tumors of the same class and grade and can be further enhanced after drug administration, as it has been observed for doxorubicin [107,340,341], which is a common substrate of P-gp, BCRP, MRP1, MRP2, MRP3 and MRP6; this complicates the development precise strategies against the tumor.

Multiple studies have proven the de novo expression of MRP3 in gliomas. In 2000, Loging et al. found an overexpression of the *ABCC3* gene in a GBM gene expression database [342]. The MRP3 protein expression in tumor samples from high-grade glioma patients was confirmed using IHC by the studies of Haga et al. (2001) [343] and Calatozzolo et al. (2005) [15], as well as in GBM cell lines by our laboratory in 2002 [337]. In 2010, Kuan et al. detected MRP3 expression by rRT-qPCR, IHC. Western blot and FACS analyzes in 90% of GBM samples analyzed [344]. In a recent meta-study, Wang et al. (2016) observed high *ABCC3* mRNA levels in GBM patients compared to normal counterparts and validated these results in GBM cell lines by RT-PCR [345]. Furthermore, in both studies, the MRP3 mRNA correlated with a higher risk of death of the GBM patients [344,345]. These results indicate that MRP3 could be used as a prognosis prediction factor.

Most tumors express *ABCB1*/P-gp in high levels [346], including schwannomas, meningiomas, low-grade gliomas (astrocytomas, pilocytic astrocytomas), and high-grade gliomas (GBMs, anaplastic astrocytomas, and anaplastic oligodendrogliomas) [66,346]. BCRP is often detected in the capillaries from brain tumors, but not in the surrounding tumor cells [79,80]. ABCB1 is the most studied ABC transporter and the principal target for transporter inhibition treatment, with the aim of increasing drug delivery [14]. Nevertheless, the importance of other multidrug-resistance related transporters has been highlighted during the last two decades.

*ABCC1*/MRP1 overexpression has been observed in several types of glioma, including GBMs, GBMs with an oligodendroglial component (GBMO) [347], anaplastic astrocytomas (grade III) [343], and meningiomas [66]. Similarly, *ABCC3*/MRP3 has been observed in high-grade gliomas such as anaplastic astrocytomas (grade III), GBM [15,343,344]. *ABCC4* and *ABCC5* mRNA overexpression and immunostaining has been observed in the glioma cells of astrocytic tumors and in the astrocytic portions of oligoastrocytomas [121]. This indicates that the expression of *ABCC4* and *ABCC5* may be associated with an astrocytic phenotype, which is probably due to its constitutive expression in astrocytes [46]. Calatozzolo et al. (2005) showed a higher expression of ABC transporters in high-grade than in in low-grade glioma using IHC. The high-grade glioma samples showed significantly higher levels of *ABCC3*/MRP3 and *ABCB1*/P-gp in the endothelial cells, but higher levels of *ABCC1*/MRP1, *ABCC3*/MRP3 and *ABCC5*/MRP5 [15].

Results from in vitro analysis should be carefully designed and evaluated. Interspecies differences in ABC transporters can be observed (Section 3.6), but culture-derived expression can be observed also. For instance, some glioma-derived cells resistant to anticancer drugs have shown to overexpress MRP2; nevertheless, MRP2 has not been detected in healthy brain or in glioma tumors neither in the mRNA nor protein level [129].

Study of ABC transporters could lead to personalized medicine strategies. It has been observed that gliomas and its surrounding tissue can present overexpression and even the de novo expression of ABC transporters [14,16,41,42,104], but they are not considered as markers for their subclassification, because there is a high variability in this phenomena, even when the same type and grade of glioma are compared [15,348]. Nevertheless, evaluation of the transporter’s expression or activity from biopsy or noninvasive methods (such as PET) could be used to devise personalized strategies for chemotherapies, such as the inhibition of ABC transporters (Section 6) [41].

### 5.5. Glioma Models to Study Drug Transport and Delivery

Drug permeability and delivery to gliomas, as well as the characteristics of the BBTB can be studied using similar methods as those described in Section 4. Some of the cancer or glioma-specific models will be briefly summarized below.

There are few in vitro models mimicking the BBTB, where the interaction of tumor cells with barrier cells can have an important impact on their function. In the specific case of gliomas, it is important to consider that the BBB and other brain barriers can be disrupted (especially in high-grade gliomas) and present a metabolic imbalance and different protein expression, as explained in Section 5.4, due to the influence of the nearby cancer cells [14,349,350]. There have been some attempts to develop BBTB models with a coculture of endothelial cells in the upper chamber of a Transwell and cancer cells in the lower chamber [350,351,352,353]. Recently, a microfluidic model was developed using human umbilical vein endothelial cells (HUVEC) and astrocytes to model the BBB or brain metastases cells to model the BBTB [354]. Comparing these models, they showed that metastases cells caused a disruption of the barrier phenotype at the BBTB, while conserving the transporter function, as observed by the P-gp efflux of accumulation of its substrate Rhodamine 123 in the luminal chamber, similarly to in vivo observations. These models can be key tools for the study of drug delivery to glioma cells across the BBB and the BBTB.

Anticancer drugs are often tested in vivo using xenograft animals or genetically engineered murine models (reviewed in [235,355]). Xenograft models are obtained by injecting with primary tumor cells or immortalized cell lines either subcutaneously or into the site of the original tumor (orthotopically) into immunocompetent or immunonaive mice or rats. This approach can be implemented easily and at relative low cost. Nevertheless, the generated tumor can misrepresent the cellular and molecular characteristics of the original tumor, due to mutations during cell passing, differences in the microenvironment of the transplanted tumor, or issues concerning the perturbed stromal setting of the immunodeficient murine host [235,356]. More recently, several distinct murine models of medulloblastoma and glioma (both oligodendroglial and astrocytic) have been developed by including into the mouse genome one or more genetic alterations previously reported to be related with the tumor formation such as mutations in the Nf1, p53 [357], kRas [358], PDGF-B [359], and GFAP [360] genes (reviewed in [235]).

Ex vivo models for the study of drugs in glioma can be obtained from dissected tumors from human patients, xenografted, or genetically modified animals to mimic the pathological conditions. As discussed in Section 4.4, tissue slices, isolated microvessels, or explants of the CP or BAB can be used for functional, physiological, drug delivery, and permeability studies, as well as for biomarkers screening [361,362,363,364,365].

### 5.6. Conclusion on the Implication of ABC Transporters in the Multidrug Resistance of Glioma

ABC transporters expressed in the blood–brain interfaces, the BBTB, and cancerous cells represent a selective barrier hindering the delivery of chemotherapeutics to gliomas and contribute importantly to their multidrug resistance, which is one of the causes of their high morbidity and mortality. In addition, the molecular heterogeneity between different grades of glioma, between patients, and even in tumor regions of a same individual, further complicate their treatment. Therefore, there have been many efforts to overcome the ABC-mediated multidrug resistance of gliomas and improve the treatment of brain cancers. Some important examples of these methodologies will be summarized in the next section.

## 6. Strategies to Improve CNS Drug Delivery in Brain Cancer

**Figure 4:**
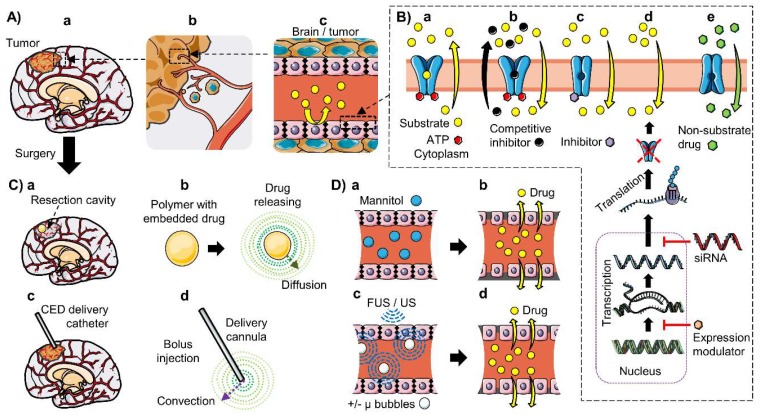
Schemas of some of the main drug delivery strategies to overcome the blood–brain barrier (BBB) and the blood–brain tumor barrier (BBTB) to treat glioma with chemotherapeutics. (**A**) (**a**) Brain gliomas may be extensively blood irrigated, (**b**) and infiltrative cells may spread to the vascularized parenchyma; (**c**) therefore, drug delivery is strongly restricted by the BBB and the BBTB. Diverse drug delivery strategies have been developed, for instance: (**B**) Modulation of the ABC transporters, (**a**) which are responsible for the brain to blood efflux of many xenobiotics, whose activity can be tuned by (**b**) competitive or (**c**) non-competitive inhibitors, or (**d**) their expression may be downregulated using siRNA or other expression modulators; (**e**) in addition, non-ABC substrates can be discovered among natural products or developed using rational design. (**C**) The BBB may be bypassed by the local delivery of chemotherapeutics; (**a** and **b**) after surgery, polymers containing an embedded drug can be implanted, (**b**) which slowly release the compound that spread by diffusion; (**c**) a catheter can be implanted to (**d**) allow the localized delivery of a chemotherapeutic compound that spread by convection. (**D**) BBB disruption can be provoked: (**a**) through osmotic disruption using a hyperosmotic product such as mannitol, (**b**) causing the shrinking of the endothelial cells and the opening of the BBB; (**c**) or through focused or unfocused ultrasounds (FUS/US) that may be aided by micro bubbles, (**d**) whose vibration breaks the tight junctions (black rhombus ♦) and allows the entry of the drug. Created using images from “smart Servier Medical Art”, Creative Commons License, 2019.

Diverse strategies have been devised to improve drug delivery into the brain for the treatment of CNS diseases (reviewed in [18,41,366]) and in the particular case of brain tumors (reviewed in [14,367,368,369]). Most of them have been concentrated in overcoming the BBB to obtain enough drug concentration for a pharmacological effect (Figure 4A). The modulation of the function or expression of ABC transporters showed promising results in preclinical studies, but it is not used in clinical due to systemic toxicity. Therefore, other methodologies have been developed, such as rationally designed drugs that are not ABC substrates; bypassing the BBB by locally delivering the drugs into the brain parenchyma and tumors; disrupting the BBB to allow the entry of chemotherapeutics; or using nanocarriers to take advantage of other transport pathways at the BBB and even target the brain or tumors. It is important to highlight that the ABC transporters can provoke the rapid clearance of drugs despite their local delivery, the disruption of the BBB tight junctions, or after drug release from nanoparticles; avoiding the chemotherapeutic to accumulate into the cancerous cells. However, interestingly, some strategies combining the use of ABC inhibitors have shown positive results.

### 6.1. Inhibition of ABC Transporters

As previously mentioned, many chemotherapeutics are effluxed by ABC transporters (Table 1; Figure 4B(a)), which are major actors of the multidrug resistance phenotype of glioma and other cancers. Therefore, there have been many efforts to improve tumor drug delivery by using competitive or non-competitive inhibitors (Figure 4B(b,c)), and many P-gp and BCRP inhibitors have been clinically evaluated for their use as adjuvants on chemotherapy to treat non-brain tumors, including valspodar, dexverapamil, tariquidar, biricodar, and elacridar; as well as indirect inhibition by anti-P-gp monoclonal antibodies [14,41,106]. Their use to overcome the BBB has also been evaluated in animals and humans (further explained in [41,57]). For instance, higher brain accumulation of erlotinib was observed when it was coadministered with elacridar to rats [273] and in a rat xenograft model of glioma [331]. In a clinical study on healthy volunteers, the penetration of ^11^C-verapamil was enhanced when coadministered with cyclosporine A [262]. However, the inhibition of ABC transporters has not been translated to clinical application due to adverse effects observed, including the cardiovascular toxicity of first-generation inhibitors [370,371] and pharmacokinetics interactions with the chemotherapeutic (e.g., the inhibition of CYPs450) leading to increased systemic cytotoxicity. In addition, many modulators inhibit more than one ABC transporter, such as elacridar and tariquidar that inhibit both P-gp and BCRP [372], which can result in other adverse effects such as the accumulation of toxic substances in brain, kidneys, liver, and other tissues.

Although the inhibition of ABC transporters itself has not proven efficiency for the clinical delivery of drugs, they have been a key resource to deepen our knowledge of the function of ABC transporters, and the multidrug resistance in cancer, glioma, and other CNS pathologies, as well as their implications in the blood–brain interfaces. As previously mentioned, (Section 4.1, Section 4.6, and Section 4.7), ABC modulators have been employed to study the function of ABC transporters in vitro, in animals and in humans. They can be co-administered with drugs to evaluate if their BBB permeability is restricted by ABC transporters, to image the transporters’ function [262], or to evaluate their substrate or inhibitor interactions with ABC transporters [130], and new inhibitors are continuously under development. Furthermore, promising results have been observed when combined with other strategies for bypassing the BBB such as convection enhanced delivery [373] (Section 6.4) and nanoparticles or targeting nanocarriers (Section 6.6) [374]. Therefore, it is worthwhile to summarize them in this section.

Many efforts have focused on the development P-gp inhibitors due to its high expression at diverse barriers of the body barriers and because it was the first ABC transporter to be associated with multidrug resistance (Section 3.2), resulting in three generations of P-gp inhibitors [41]. The first generation was composed mainly of repurposed drugs, such as verapamil, cyclosporine A, and tamoxifen. These acted as competitive inhibitors (Figure 4B(b)), requiring the administration of high doses [375] and thus leading to cardiovascular toxicity in vivo [370,371] and pharmacokinetics interactions between the chemotherapeutic and the modulator [370]. Second-generation modulators were developed by modifying the structure of first-generation molecules to improve specificity and potency, achieving reduced systemic toxicity, including dexverapamil, biricodar citrate (VX-70), dexniguldipine, and valspodar (PSC-833). Some of these molecules arrived at clinical trials, such as the cyclosporine D derivative valspodar (PSC-833), which inhibits P-gp 10–20 times more than cyclosporine-A. Nevertheless, it was observed that these chemosensitizers could also inhibit CYPs450 enzymes, inducing the higher toxicity of the coadministered agents in other tissues and cells [41]. Then, third-generation P-gp modulators were designed using the structure–activity relationship and combinatorial chemistry, achieving a 300-fold improvement in inhibition potency with an effective function at the nanomolar range while minimizing pharmacokinetic interactions due to a reduced cross-inhibition of other ABC transporters and CYPs450. This group includes tariquidar (XR-9576), elacridar (F12091), laniquidar (R101933), zosuquidar (LY.336979), and diarulimidazole (ONT-093). For instance, tariquidar showed an improved permeation of sunitinib, sorafenib, dasatinib, or temozolomide and veliparib [14].

BCRP and the MRPs can be inhibited by P-gp modulators or specific inhibitors. For instance, the structurally related P-gp inhibitors elacridar and tariquidar are also BCRP inhibitors [372]. BCRP-specific inhibitors contain more nitrogen atoms and aromatic moieties than those shared with P-gp [53] and include, for instance, natural substances as fumitremorgin C (FTC) [376] and its less toxic and more active tetracyclic analog Ko143 [377]. Interestingly, tyrosine kinase inhibitors such as gefitinib, erlotinib, and imatinib (Gleevec) can block the ATPase activity of BCRP, while also having a chemotherapeutic effect [378], and they could be used as both inhibitors and effectors. The MRP family also presents specific inhibitors. For instance, MRP1-3 are inhibited by several non-nucleoside and nucleoside reverse transcriptase inhibitors used as anti-HIV-drugs, but specially tenofovir, delavirdine, efavirenz, and emtricitabine [379]. MRP4 is inhibited by the anti-inflammatory molecules celecoxib and diclofenac, while MRP5 function is altered by phosphodiesterase inhibitors zaprinast and trequinsin [112,380].

### 6.2. Other Modulators of ABC Transporter-Dependent Multidrug Resistance

Another strategy to modulate ABC function that is being investigated in vitro and in vivo, but still not in clinical assays, is the downregulation of their expression using either small xenobiotics, natural products or small interfering RNAs (siRNAs) (Figure 4B(d)). For instance, Tanshinon II-A from *Salvia miltiorrhiza* downregulated the expression of P-gp, MRP1, and BCRP in dox-resistant breast cancer cells (MCF/dox), improving Dox sensitivity [381]. Recently, it was reported that Fasudil (HA-1077), an inhibitor of Rho-associated protein kinases (ROCKs) used in China and Japan for the treatment of cerebral vasospam, increases the temozolomide (TMZ) sensitivity of TMZ-resistant gliomas in vitro and in xenografted mouse and rat by suppressing the expression of BCRP through the ROCK2/moesin/β-catenin pathway [382]. Recently, siRNAs have emerged as tools to selectively downregulate protein expression using a double-stranded RNA of between 21 and 28 nucleotides that selectively blocks and induces the degradation of a specific mRNA [383]. It has been observed that blocking *ABCB1* or *ABCG2* with the specific exogenous siRNAs can reverse multidrug resistance in diverse cancer cells [384,385]; even more, the concomitant application of both siRNAs using a nanoparticle-facilitated delivery showed a synergic effect in breast cancer cells [386].

Other natural products and xenobiotics have shown to diminish the multidrug resistance phenotype, although the modulation mechanism (inhibition or expression) has not been elucidated for all of them, such as polyphenols, flavonoids, and stilbenes from Chinese plant extracts [387]. Furthermore, trabedectedin, halaven, and cytarabine reverse multidrug resistance and have been recently accepted for clinical use [388,389]. Interestingly, JL-17 presents a higher inhibition of P-gp than verapamil, increasing anticancer drug accumulation in K562/A02 cells [390]. These molecules have not been tested in the case of glioma drug delivery, but they could be an interesting alternative.

### 6.3. Rational Drug Design

Rational drug design to obtain BBB-permeable molecules can achieve improved efficacy (Figure 4B(e)). Using in silico tools (Section 4.8), existing drugs can be improved, or chemical libraries can be screened, to obtain molecules that are able to pass through the BBB [289,290,391]. For instance, Salphati et al. used a central nervous system multiparameter optimization (CNS-MPO) model to improve the physiochemical properties of PI3K inhibitors and developed two molecules, GNE-317 and GDC-0084, with improved BBB penetration and tumor distribution and growth inhibition in xenograft mice [392,393,394]. Although a later study did not observe an improved survival in a murine glioma model compared to GDC-0980, a PI3K inhibitor with low brain penetrance [395], GDC-0084 is currently in phase II clinical trials for newly diagnosed GBM (ID NCT03522298 [396]).

### 6.4. Local Delivery: Polymeric Drug Delivery Systems and Convection-Enhanced Delivery

Other methodologies have been devised to locally deliver chemotherapeutics into the brain parenchyma or the tumor aiming to bypass the BBB, avoiding the need to overcome the ABC transporters acting as gatekeepers and diminish the systemic toxicity (Figure 4C). These methodologies depend on the effective diffusion of the compound through the tumor; thus, lipophilic molecules that can passively enter the tumor cell membranes are used. Nevertheless, although they are still subject to the CNS clearance mechanisms, the drugs can be rapidly ejected into the bloodstream by the BMVEC through passive transport or active efflux, significantly reducing the volume of distribution of the molecules in the brain [367].

Polymeric drug delivery systems containing a chemotherapeutic molecule can be implanted during surgery and locally release the drug for a prolonged time (Figure 4C(a,b)). Biodegradable hydrophobic polymers that slowly degrade after placement into the brain are used to release the embedded drug in a nearly constant rate (reviewed in [397]). Gliadel wafers are small implantable polymer wafers that are placed into the tumor-resection cavity and locally deliver over several days or weeks the encased carmustine (BCNU), which is a nitrosourea that is used to treat GBM and other tumors [398,399,400]. These devices were approved by the FDA in 1996 for the treatment of recurrent GBM and in 2004 for primary GBM, but they are not widely used because of the need of trained surgeons for their implantation, possible complications, and high cost of therapy regarding a small prognosis improvement [367,401]; indeed, patient trials have shown only a modest increase in survival of 8 to 9 weeks compared to a placebo [398,399]. In addition, carmustine is a small lipophilic molecule that is not effluxed by ABC transporters, but it passively diffuses into systemic circulation rapidly upon release from the wafers [397]. Nevertheless, there is a continuous development on these devices, searching to improve their efficiency [397].

Convection-enhanced delivery (CED) uses catheters implanted during surgery to constantly deliver chemotherapeutics into the tumor area, where the drug is supposed to reach all the tumor cells via convective flow due to the hydrostatic pressure gradient created [402,403] (Figure 4C(c,d); reviewed in [404,405]). Nevertheless, depending on the compound, the tissue around the catheter may receive enough drug, but its concentration may rapidly decrease in nearby zones due to efflux into the vessels [367,406]. The co-infusion of imaging tracers allows the real-time tracking of the convective infusate flow to evaluate the volume effectively targeted with the chemotherapeutic compound [407,408]. Although there have been several phase I and phase II clinical trials using CED, they have not shown a positive effect on patient outcomes [404]. A phase III trial where CED was used to administrate interleukin-13 bound to *Pseudomonas* exotoxin to GBM patients did not show a significant improvement compared to Gliadel wafers [409]. Nevertheless, other preclinical studies have shown promising results [410], and continuous efforts have been made to improve the efficiency of CED, including works in catheter technology and optimal positioning [406]. Recently, a chronic CED administration system was used to infuse the topoisomerase inhibitor topotecan in a pig model for up to 32 days without toxicity [411]. The use of ABC inhibitors has been suggested as a strategy to improve CED and could be further explored. For instance, a recent study showed an improvement of tumor apoptosis in a transgenic H3.3K27M mutant murine model of diffuse intrinsic pontine glioma by a pretreatment of dexamethasone plus tariquidar before convection-enhanced delivery (Section 6.5) of dasatinib [373].

### 6.5. BBB Disruption: Osmotic Disruption and Ultrasound-Enhanced Delivery

Osmotic disruption of the BBB can be achieved by infusing a hyperosmotic compound such as mannitol into the carotid artery through a catheter [412], which causes the endothelial cells to shrink, disrupting the tight junctions and opening the paracellular space, followed by the administration of a chemotherapeutic (Figure 4D(a,b)). The first clinic assays using this method in six patients with malignant brain tumors showed an increase in methotrexate tumor delivery, overcoming the ABC transporters mediated efflux of the chemotherapeutic. It has been used for the treatment of chemosensitive primary CNS lymphoma (PCNSL), showing a prolonged response without using radiotherapy in a multicenter clinical trial between 1982 and 2005 [413]. The use of intraarterial administration of bevacizumab after osmotic BBB disruption with mannitol has shown an increase in progression-free survival in GBM patients in phase I trials [414,415] and is undergoing phase II trials (ID NCT01238237 [416]). Nevertheless, opening the BBB is not without risk, as it has been observed that it can increase the risk of other neurological problems, such as edema, stroke, and epileptic seizures [414,417].

Focused ultrasound-enhanced delivery (FUS) methods use directed low-frequency ultrasound waves to temporally open the tight junctions between the endothelial cells [418] (Figure 4D(c,d)). This has been employed to enhance the delivery of BBB-impermeable liposome-encapsulated doxorubicin in a rat glioma model [419]. Disruption of the BBB with FUS can have harmful effects such as intracerebral hemorrhage, erythrocyte extravasation, and edema [420]. Thus, there are continuous efforts to diminish these effects, such as microbubble (MB)-enhanced (Figure 4D(c)) FUS and the use of sonosensitizers that provoke a localized cytotoxic effect. The oscillation of MBs stimulated by ultrasounds provoke a mechanical disruption of the BBB at lower frequencies of ultrasounds than FUS alone; for instance, this strategy has been employed to improve carmustine (BCNU) penetration into the rat brain [421]. Sonodynamic therapy using the 5-aminolevulinic acid (5-ALA) has been combined with transcranial MRI-guided focused ultrasound (MRgFUS) and real-time MRI thermometry to monitor and optimize the therapy in a rat brain tumor model, achieving an improved survival time [422]. Nevertheless, in a recent study, physical BBB disruption provoked by MB-enhanced FUS did not impact the brain kinetics of ^11^C-erlotinib, while elacridar did increased its brain penetration (with or without FUS), indicating that erlotinib delivery into the brain is governed by ABC transporters efflux and not by the physical integrity of the brain and suggesting that the selection of the chemotherapeutic for FUS is critical, as this strategy may not overcome the ABC-mediated efflux [273].

After obtaining positive results in rabbit and primates [423,424], an implantable unfocused ultrasound device has undergone a phase I/IIa clinical trial (ID NCT02253212 [425]) with 19 patients of recurrent GBM who received 4 to 16 min of low-intensity pulsed ultrasounds for BBB disruption followed by IV carboplatin chemotherapy every four weeks, during one to 10 sessions. Although one patient presented a transient edema, the results were promising with no carboplatin-related neurotoxicity, progression-free survival of 2.73 months, and a median overall survival of 8.64 months [426].

### 6.6. Nanoparticles and Targeting Nanocarriers

Nanoparticles can be used to entrap or encapsulate chemotherapeutics drugs aiming to improve their BBB permeability and specifically target the BBB or brain tumors through specific ligands. These nanocarriers can present diverse chemistries, including the material and surface functionalization strategies; the characteristics, studies, advantages, and limitations of nanocarriers are extensively reviewed in this special issue by Teleanu et al. [427]. For instance, diverse reports use polymeric nanoparticles, solid lipid nanoparticles, liposomes, dendrimers, micelles, inorganic nanoparticles, carbon nanotubes, and quantum dots. Depending on this chemistry and their functionalization, they may pass through the BBB by diverse pathways, mainly: paracellular, transcellular, or carrier-mediated transport; and receptor-mediated or adsorptive transcytosis [427]. They can be functionalized, for instance, with ligands to cross the BBB via receptor-mediated transcytosis (such as glutathione) or more specific ligands to target tumor cells, such as PEG and glioma homing peptides [428], as well as sialic acid, glucosamine, and concanavalin A, which have shown higher brain accumulation of paclitaxel in rats [429].

Some nanoparticles have shown promising results in preclinical and clinical studies for the treatment of brain tumors. For instance, PEGylated liposomal doxorubicin showed higher brain penetration than free doxorubicin in an intracranial model of breast cancer brain metastasis mouse model [430]. Glutathione pegylated liposomal doxorubicin (2B3-101) [430] showed higher penetration into the brain of a murine model and underwent a phase I/IIa clinical study, obtaining a progression free survival of three months in 58% of treated patients of breast cancer brain metastasis [431]. In addition, studies with micellar formulations for the delivery of curcumin [432] and multi-walled carbon nanotubes [433] have shown increased cancer cell uptake in vitro and brain tumor penetration in in vivo assays. Once the drug is released, it may be subjected to passive or active transport mechanisms; thus, some studies have evaluated the use of multi-compound nanoparticles to overcome the multidrug resistance in cancer (reviewed in [374]). For instance, doxorubicin-curcumin nanoparticles have proven an increased accumulation in vitro [434] and in vivo in cancer preclinical models, which has been accompanied by a reduced cardiotoxicity [435]. Similarly, a liposomal cocktail including a pH-responsive molecule (i.e., malachite green carbinol base (MG)) and liposome conjugated with Her-2 antibody has been employed for the codelivery of doxorubicin and verapamil, overcoming the doxorubicin resistance in vitro and enhancing tumor inhibition in a xenografted mouse model of breast cancer [436]. Although nanoparticles codelivering a chemotherapeutic and an ABC inhibitor have not been tested in human patients nor in glioma models, this could be an interesting approach to treat CNS cancers.

### 6.7. Conclusion on Strategies to Improve CNS Drug Delivery in Brain Cancer

Many of the strategies and technologies for the delivery of medicines into the CNS and glioma have focused on bypassing the BBB and the ABC transporter mediated efflux of chemotherapeutics. Few of them have been approved by regulating agencies such as the FDA and EMA because of systemic toxicity or insufficient improvements in prognosis; nevertheless, they are constantly under development. In addition, there have been promising results with new technologies such as ultrasound-mediated BBB disruption and nanoparticles. It is important to highlight that ABC transporters may drive the rapid clearance of their drug substrates despite BBB disruption or localized delivery into the parenchyma. Thus, it would be recommendable to combine these technologies with rationally designed drugs that are not effluxed by ABC transporters or by the coadministration of modulators, which has also shown positive results.

## 7. Conclusions

It is fundamental to consider the barrier function of the blood–brain interfaces during the development of CNS therapeutics or drug brain-delivery strategies. Importantly, the ABC transporters found in the blood–brain barrier (BBB), the blood–cerebrospinal fluid barrier (BCSFB), the arachnoid barrier (BAB), and the blood–brain tumor barrier (BBTB) regulate the exchange of a wide variety of molecules between the blood and the brain parenchyma. Additionally, ABC transporters expressed in glioma cells constitute a second barrier against chemotherapeutics.

A large diversity of drug delivery strategies has been developed with promising results at the preclinical stage, but many have not shown an important improvement in the clinical fight against brain tumors, especially for high-grade gliomas such as glioblastoma multiforme. Considering the delicate homeostasis of the brain and the systemic importance of ABC transporters, special care should be taken when designing chemotherapeutic drugs, ABC modulators, or strategies to disrupt or circumvent the BBB. Therefore, deepening our knowledge on the blood–brain interfaces and particularly on ABC transporters’ function and expression is of extreme utility, not only to better understand the multidrug resistance phenomena, but also to develop better strategies to improve drug delivery.

## Figures and Tables

**Table 1:** Classes of substrates and examples of chemotherapeutics transported by drug-related ABC transporters.

Gene; Protein	Substrates Classes	Examples of Chemotherapeutics Substrates
*ABCB1*; P-gp MDR1 [16,42,50,51]	Amphipathic cations, organic molecules. No structure–activity relationship has been identified	Alkylating agents: temozolomide (TMZ) *, procarbazine *, carmustin * Topoisomerase inhibitors: etoposide *, topotecan *, irinotecan *, teniposide, doxorubicin, daunorubicin, carboplatin *, mitoxantrone Tyrosine kinase inhibitors: erlotinib *, dasatinib *, sunitinib *, sorafenib *, imatinib mesylate, gefitinib Anti-microtubule taxanes: paclitaxel *, docetaxel Dihydrofolate reductase inhibitor: methotrexate Vinca alkaloids: vinblastine *, vincristine * PARP1/2 inhibitor: veliparib (ABT-888) * MGMT inhibitor: lomeguatrib (O^6^Benzylguanine/O^6^BG) *
*ABCG2*; BCRP [16,42,52,53,54]	Partial overlap with P-gp substrates	Alkylating agents: temozolomide (TMZ) * Topoisomerase inhibitors: etoposide *, topotecan *, mitoxantrone, irinotecan *, SN-38, 9-aminocamptothecin, doxorubicin Tyrosine kinase inhibitors (TKI): erlotinib *, dasatinib *, sunitinib *, sorafenib *, imatinib, gefitinib, nilotinib PARP1/2 inhibitor: veliparib (ABT-888) * MGMT inhibitor: lomeguatrib (O^6^Benzylguanine/O^6^BG) *
*ABBC1*; MRP1 [16,55,56,57]	Organic anions, glutathione conjugates. Glutathione (GSH)-dependent	Alkylating agents: cyclophosphamide Topoisomerase inhibitors: doxorubicin, etoposide *, campathecin, camptothecin, irinotecan * (CPT-11) Anti-microtubule taxanes: paclitaxel * Dihydrofolate reductase inhibitor: methotrexate Vinca alkaloids: vinblastine *, vincristine *
*ABCC2*; MRP2 [16,55,56,57]	Organic anions, glutathione conjugates. Allosteric stimulation by bile acids, sulfinpyranzone, penicillin G, and indomethacin; but not GSH	Alkylating agents: chlorambucil, cyclophosphamide, cisplatin *, oxaliplatin Topoisomerase inhibitors: doxorubicin, etoposide *, epirubicin mitoxantrone, irinotecan *, glucuronidated SN-38 Vinca alkaloids: vinblastine *, vincristine * Antineoplastic, dihydrofolate reductase inhibitor: methotrexate Antineoplastic, angiotensin inhibitors: valsartan, olmesartan
*ABCC3*; MRP3 [16,55,56,57]	Organic anions, glutathione conjugates. Not stimulated by GSH nor bile acids	Alkylating agents: cisplatin * Antineoplastic, dihydrofolate reductase inhibitor: methotrexate Topoisomerase inhibitors: etoposide *, teniposide, doxorubicin Vinca alkaloids: vincristine * Conjugates: dinitrophenyl S-glutathione, acetaminophen glucuronide
*ABBC4*; MRP4 [16,55,56,57]	Organic anions, glutathione conjugates, cyclic nucleotides. GSH requirement depending on substrate; but not for cAMP or cGMP	Antineoplastic, dihydrofolate reductase inhibitor: methotrexate Topoisomerase inhibitors: topotecan * Nucleotide analogues: 6-mercaptopurine, 6-thioguanine
*ABBC5*; MRP5 [16,55,56,57]	Organic anions, glutathione conjugates, cyclic nucleotides. GSH requirement not exactly established, depending on substrate; but not for cAMP or cGMP	Antineoplastic, dihydrofolate reductase inhibitors: methotrexate Platinum-based drugs: cisplatin * Nucleotide analogues: 6-mercaptopurine, 6-thioguanine Conjugates: dinitrophenyl S-glutathione Heavy metals: cadmium chloride, potassium antimonyl tartrate
*ABCC6*; MRP6 [16,55,56,57]	Organic anions, glutathione conjugates. GSH requirement not stablished	Alkylating agents: cisplatin * Topoisomerase inhibitors: etoposide *, doxorubicin, daunorubicin

* Reported use in glioblastoma multiforme (GBM) [16,42]; PARP(1/2): Poly(ADP-ribose) polymerase (1/2); MGMT: O^6^−methylguanine methyltransferase.

**Table 2:** Subcellular localization and level of evidence (protein and/or mRNA) of ABC transporters at the brain barriers of humans and rodents under non-pathological conditions.

Gene; Protein	BBB	Parenchymal Cells	BCSFB	AB
*ABCB1*; P-gp/MDR1 *Abcb1a* and *Abc1b* Mdr1a and Mdr1b (r, m)	Luminal: h, r (Mdr1a), m (Mdr1a) mRNA and protein: h, r (*Abcb1a*), m (*Abcb1a*) [22,58,59,60,61,62,63]	Not detected in healthy tissue (h, r, m) [64,65,66,67,68,69]	Apical: h, r, m mRNA and protein: h, r (*Abcb1a, Abcb1b*), m (*Abcb1a*) [70,71,72,73]	Apical: h, r, m mRNA and protein: h, r, m [33,34,74,75,76,77]
*ABCG2;* BCRP	Luminal: h, r, m mRNA and protein: h, r, m, p [22,63,78,79,80,81,82,83]	Unclear mRNA and protein: Neuropil (h); cultured astrocytes (h, r) mRNA: Microglia (h, m) [65,68,84,85]	Apical: h, m mRNA and protein: h, r, m [20,33,70,81]	Apical: h, r, m mRNA and protein: h, r, m [33,34,74,75,76,77]
*ABBC1*; MRP1	Luminal: h * protein: h * mRNA: h *, r, m, c (low) [46,86]	Not detected [64]	Basolateral: h, r, m Protein and mRNA: h, m, r [70,71,73,83,87,88,89]	mRNA: h, r, m Protein: r [33,34]
*ABCC2*; MRP2	Luminal: r, m protein: r, m mRNA: r (low), m, c (low) [46,86,90,91]	mRNA and protein: neuropil, glial and neuronal cells (h) [64]	mRNA: h, r [87,88]	Not detected (h, r, m) [33,34]
*ABCC3*; MRP3	mRNA: h * (low), r (low), m [46,86]	Not detected [64]	mRNA: h, r [87,88]	Not detected (h, r, m) [33,34]
*ABBC4*; MRP4	Luminal: h, r, m Protein: h, r, m mRNA: h, r, m [22,46,86,92,93,94,95]	Not detected [64]	Basolateral: h, r, m Protein & mRNA: h, r, m [83,87,88,96]	mRNA: h, r, m Protein: r [34]
*ABBC5*; MRP5	Luminal: h, r, m mRNA: h, r, m [46,86]	mRNA & protein: Neuropil (h) [64]	Basolateral: r mRNA & protein: h, r [83,87,88]	Not detected (h, r, m) [33,34]
*ABCC6*; MRP6	mRNA: h *, r, m [46,86]	Not analyzed [64]	mRNA: h, r [87,88]	mRNA & protein: r [34]

h: human, r: rats, m: mice, p: porcine; * only in samples from diseased patients.

**Table 3:** ABC transporters expression in human brain tumors the brain and gliomas.

Gene; Protein	Location in Human BRAIN Tumors
ABCB1; P-gp/MDR1	Tumor capillaries; schwannomas, gangliogliomas, meningiomas, low-grade gliomas (astrocytomas, pilocytic astrocytomas) and high-grade gliomas (glioblastoma multiforme (GBM), anaplastic astrocytomas and anaplastic oligodendrogliomas)
ABBC1; MRP1	Tumor capillaries, glioma cells, neuronal components of gangliosomas
ABCC2; MRP2	ND
ABCC3; MRP3	Anaplastic astrocytomas (grade III), GBM; cultured cancer and ECs from GBM
ABBC4; MRP4	Tumor capillaries; astrocytic tumors; and astrocytic portions of oligoastrocytomas
ABBC5; MRP5	Tumor capillaries; astrocytic tumors; and astrocytic portions of oligoastrocytomas
ABCC6; MRP6	NAn
ABCG2; BCRP	Tumor capillaries; ND in glioma cells in situ

ECs: endothelial cells; ND: Not detected; NAn: not analyzed/no data available.
